# Dissecting *Leishmania infantum* Energy Metabolism - A Systems Perspective

**DOI:** 10.1371/journal.pone.0137976

**Published:** 2015-09-14

**Authors:** Abhishek Subramanian, Jitesh Jhawar, Ram Rup Sarkar

**Affiliations:** 1 Chemical Engineering and Process Development, CSIR-National Chemical Laboratory, Pune, Maharashtra, India; 2 Academy of Scientific & Innovative Research (AcSIR), CSIR-NCL Campus, Pune, India; King's College London, UNITED KINGDOM

## Abstract

*Leishmania infantum*, causative agent of visceral leishmaniasis in humans, illustrates a complex lifecycle pertaining to two extreme environments, namely, the gut of the sandfly vector and human macrophages. *Leishmania* is capable of dynamically adapting and tactically switching between these critically hostile situations. The possible metabolic routes ventured by the parasite to achieve this exceptional adaptation to its varying environments are still poorly understood. In this study, we present an extensively reconstructed energy metabolism network of *Leishmania infantum* as an attempt to identify certain strategic metabolic routes preferred by the parasite to optimize its survival in such dynamic environments. The reconstructed network consists of 142 genes encoding for enzymes performing 237 reactions distributed across five distinct model compartments. We annotated the subcellular locations of different enzymes and their reactions on the basis of strong literature evidence and sequence-based detection of cellular localization signal within a protein sequence. To explore the diverse features of parasite metabolism the metabolic network was implemented and analyzed as a constraint-based model. Using a systems-based approach, we also put forth an extensive set of lethal reaction knockouts; some of which were validated using published data on *Leishmania* species. Performing a robustness analysis, the model was rigorously validated and tested for the secretion of overflow metabolites specific to *Leishmania* under varying extracellular oxygen uptake rate. Further, the fate of important non-essential amino acids in *L*. *infantum* metabolism was investigated. Stage-specific scenarios of *L*. *infantum* energy metabolism were incorporated in the model and key metabolic differences were outlined. Analysis of the model revealed the essentiality of glucose uptake, succinate fermentation, glutamate biosynthesis and an active TCA cycle as driving forces for parasite energy metabolism and its optimal growth. Finally, through our *in silico* knockout analysis, we could identify possible therapeutic targets that provide experimentally testable hypotheses.

## Introduction

*Leishmania infantum* is the causal agent of visceral leishmaniasis, a systemic disease that is transmitted by female phlebotomine sandflies [1]. The disease is believed to claim around 50,000 human lives and around half a million cases of visceral leishmaniasis are being reported every year [2]. The parasite demonstrates a digenetic lifecycle; the motile promastigote forms that survive and proliferate in the midgut of the sandfly vector, and the amastigotes that lasts in the phagolysozome of the macrophages of the human host. The sandfly gut is mildly alkaline and abundant in glucose while the phagolysozome is highly acidic and scarce in glucose [3–5]. These extreme environments direct the parasite to dynamically vary its metabolism, for efficient adaptation and survival. The *Leishmania* parasites have been extensively scrutinized for the carbon sources that it prefers during its lifecycle [6]. But, information related to *Leishmania* amastigote metabolism is more or less fragmented and incomplete [7]. Also, a large scale unified analysis to identify the metabolic routes by which *L*. *infantum* achieves in both the physiological states still needs to be accomplished. With the aid of transcriptomic and proteomic analyses, it is speculated that certain metabolically crucial enzymes display characteristic stage-specific regulation, both in mRNA and protein levels aiding the parasite in adapting to changing environments [3, 5, 8–10]. The impact of these changes on the actual metabolic reactions at the pathway level and hence, the effect on parasite survival requires further exploration. The identification and targeting of these reactions essential for parasite survival might hence, lead to development of better treatment strategies and eradicate parasite infection.

Sequencing of parasite genomes has paved way to the reconstruction of metabolic pathways from genome data [11]. Metabolic pathway reconstruction essentially includes the identification of enzymes catalyzing metabolic reactions. These enzymes are associated to their appropriate subcellular locations by analyzing the information essentially obtained both from heterogeneous experiments and gene/protein sequence analysis. For large scale metabolic reconstructions, where availability of kinetic data for numerous reactions is limiting, the reconstruction can be represented as a constraint-based model and used for analysis [12, 13]. The reconstructed network can be analyzed by defining biological constraints to the reactions considered. These models can be studied through specific methods, such as flux balance analysis (FBA), leading to both qualitative and quantitative predictions about the flux distribution [14]. These predictions may be further analyzed through experimental investigations.

Previously, a number of constraint-based models have been published on Trypanosomatids [15–17]. Specifically for *Leishmania*, a genome-scale model of *L*. *major* metabolism was reconstructed using heterogeneous information and was analyzed using flux balance analysis to understand whole cell metabolism in the cutaneous species and predict genes essential for its growth [17]. Glucose and certain non-essential amino acids have been shown to be utilized in both the life stages of *Leishmania* species and to be essential for either survival or virulence [18, 19]. Additionally, most enzymes of the glucose catabolism have been shown to be essential for either survival or virulence of trypanosomatids [20–24]. Therefore, we wanted to study glucose metabolism, energy metabolism, non-essential amino acid metabolism and their interplay in promoting growth of *L*. *infantum* parasite. With the objective to study the energy metabolism of the visceral species *Leishmania infantum*, we here *de novo* reconstructed a constraint-based model iAS142 that accommodates the pathways related to glucose metabolism, energy metabolism and biosynthesis of non-essential amino acids. The iAS142 model encompasses a total of 237 total reactions, out of which 115 reactions could be associated with a total of 142 genes. The reactions were distributed across 5 different model compartments: four subcellular–the glycosome, the mitochondrion, the mitochondrial intermembrane space and the cytosol, and an extracellular for exchange of metabolites. We adopted a similar strategy for metabolic reconstruction as other reconstruction studies [25]. But, we extended this strategy for further confirmation of reaction subcellular locations through comparisons with homologous sequences. Most importantly, in our study, we also attempted to assign genes to some transport reactions and also identify novel non-gene associated transports from literature.

From our model, we explored many scenarios of parasite growth and attempted to capture the changes in energy metabolism with respect to the changing environmental conditions. To analyze the model, we presented a novel biomass equation from formerly published 13C isotopic enrichment data [19]. The biomass reaction actually signifies a metabolic demand reaction devoid of ATP that represents the drain of specific metabolites considered in the model. We demonstrated that the formulated biomass reaction is sufficient to maintain the cellular energy requirements, as it is coupled to the ATP maintenance reaction (considered as a separate reaction in the model) through redox balance. Using this reaction, we validated the model through i) typical reaction knockout analysis, where the growth phenotype information predicted through the model was compared with the information available through knockout literature obtained for *Leishmania* species; and also by ii) enumerating model conditions so as to capture the secretion of experimentally observed overflow metabolites in *Leishmania*. We compared our *Leishmania* specific biomass objective function with the biomass previously used in the iSR215 *Trypanosoma cruzi* constraint-based FBA model [15] and demonstrated that the choice of the biomass objective function to a large extent affects the model predictions. Also from the model, effect of amino acid uptake on energy metabolism, when supplemented with glucose, was systematically investigated. Notably, the scenarios for the promastigote and amastigote parasite forms were created in the model, and the differences in preferred metabolic routes, required for enhanced adaptation in the variable conditions, were predicted.

As a part of rational drug design in *Leishmania* species, identification of novel drug targets in an early discovery phase has become increasingly important so as to design new small molecule inhibitors that can serve as potential drug candidates against the parasite [22, 24]. The amastigote developmental stage is the most sought after for drug discovery as this stage is virulent in humans. Though, a number of drug targets in *Leishmania* have been proposed previously, only few of them could be validated through experiments [24]. Through the reaction knockout analysis carried out in our model, the genes governing lethal phenotypes were deemed to be potential chemotherapeutic targets. Although, one might get a qualitative understanding by performing a standard knockout study in a metabolic network, a quantitative reconciliation would provide a better understanding of controlling the predicted drug targets. Hence, the essential reactions predicted from reaction knockout analysis were further used to simulate chemotherapeutic intervention scenarios to abolish growth in the model-presumed amastigote stage.

Thus, our simple modeling approach could reveal many important cellular consequences of the *Leishmania infantum* energy metabolism substantiating the strength of suitable *in silico* metabolic reconstruction strategies in creating biologically feasible predictions. Additionally, the model reaction knockout analyses along with simulation of chemotherapeutic interventions give a larger applicability of a constraint-based model in predicting and categorizing chemotherapeutic targets.

## Methods

### Model reconstruction

**Fig 1:**
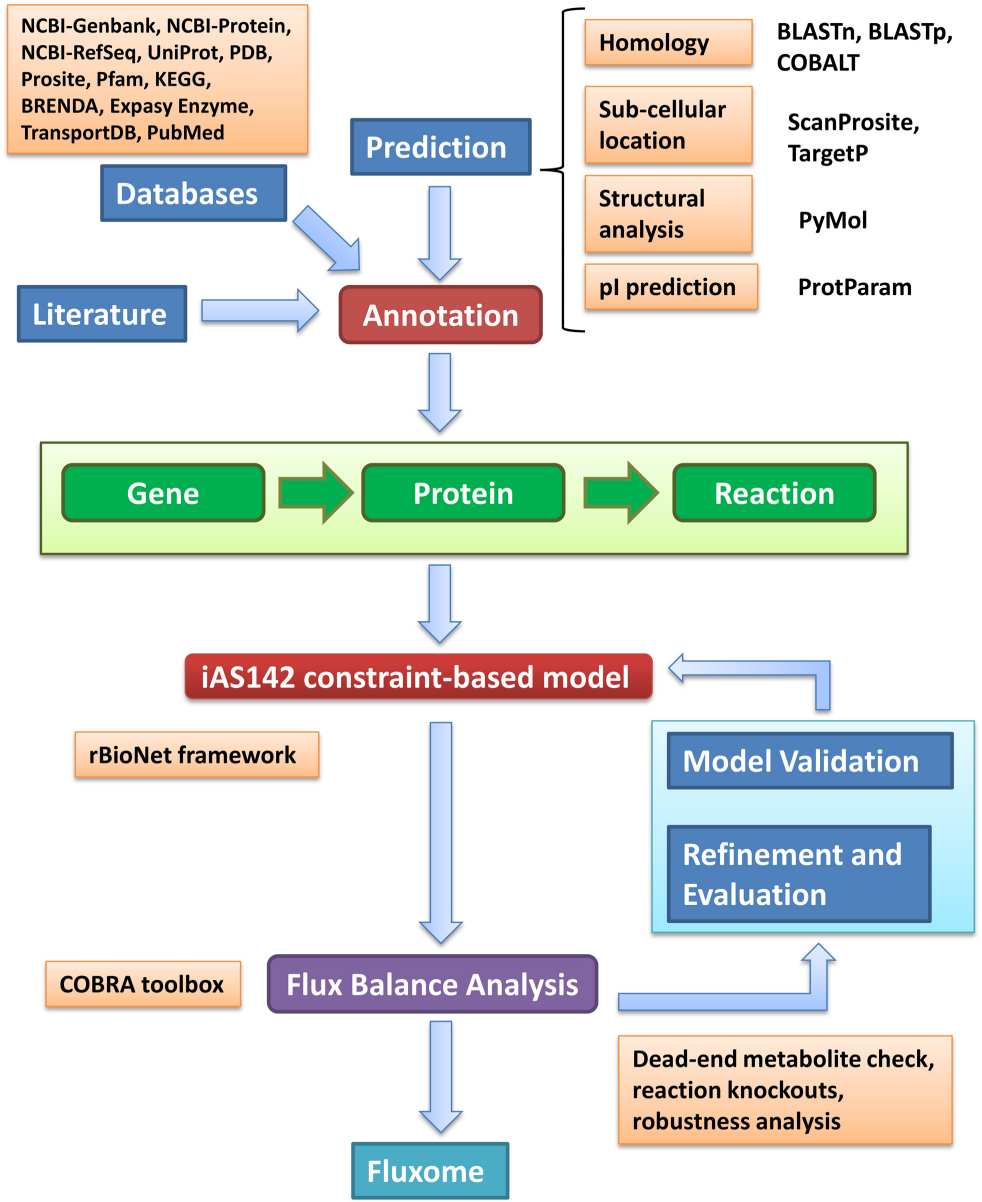
Strategy for reconstruction of the *Leishmania infantum* constraint-based model, iAS142.

Using information from heterogeneous sources, metabolic reconstruction essentially includes the identification and association of enzymes to crucial metabolic reactions and their assignment to appropriate subcellular locations [11]. Integrating this heterogeneous information, a gene-protein-reaction (GPR) framework is created with respect to cellular compartments and is used for large scale analyses and predictions. As an attempt to understand energy metabolism in *Leishmania infantum*, here we reconstructed the GPR considering the reactions involved in energy generation (ATP) and production of critical metabolites by following a stringent reconstruction strategy considering heterogeneous data, tools and supporting analyses (Fig 1). A list of reactions, their corresponding genes, enzymes, and metabolites was compiled with appropriate literature support, in combination with sequence analysis to substantiate evidence related to reaction, its biological significance, and location (S1 Table). The metabolites and the corresponding reactions considered were distributed into 5 different model compartments—glycosome, cytosol, mitochondrion, mitochondrial inter-membrane space and extracellular. All the above information was organized in the rBioNet toolbox [26] to eventually get an extensive model reconstruction iAS142 for *L*. *infantum* energy metabolism. The model is available in the SBML format, iAS142_recon.xml, for further reference (S1 File).

### Improving annotations

The genes considered for model reconstruction were obtained for *Leishmania infantum* JPCM5 genome (assembly ID: GCA_000002875.2 ASM287v2) updated as of 16/12/2011. The reactions considered in the model and the corresponding Enzyme Classification numbers (E.C Nos.) were curated from databases like BRENDA [27], Expasy Enzyme [28], KEGG [29]. The research articles referred in order to curate the various reactions in the network are enlisted in S1 Table alongside the corresponding reactions. Every reaction considered in the network was associated with a gene present in the genome of *Leishmania infantum*. Each enzyme was annotated for its molecular function, its associated pathway, and its subcellular location within the cell.

The molecular function of each protein was annotated through primary sequence search against databases like NCBI GenBank [30], UniProt [31], and KEGG [29]; motif/domain search against domain databases, like Pfam [32], and Prosite [33]; and manual curation through literature survey. Also, function of glucose-6-phosphate epimerase (previously annotated as aldose-1-epimerase) was confirmed through structural analysis to identify conserved stretches of important active site residues of these protein sequences by comparing them with the 3D structures of homologues acquired from the Protein Data Bank (PDB) [34]. Structural analysis was carried out in PyMol version 1.4.1 [35] to find the active site residues in the PDB structure that interact with the substrate (see Section A in S1 Text for more details).

Most of the reactions were assigned to a particular cellular compartment on the basis of direct literature evidence, which could suggest the subcellular location of the enzyme catalyzing that particular reaction. Reaction information from *Leishmania* and other Trypanosomatids were referred for curating the subcellular location of the enzymes considered in the model (see S1 Table). Hence, along with the literature evidence, to affirm the subcellular location of the enzyme specific to *Leishmania infantum*, the corresponding protein sequence was also used for sequence based detection of subcellular targeting signals that might predict the intracellular location of that particular enzyme. The enzymes of *L*. *infantum* genome for which literature evidence (indicating its subcellular location) was unavailable, subcellular location was explicitly assigned by *in silico* identification of subcellular localization signals within each protein sequence. TargetP was used for predicting the presence of a mitochondrial target peptide within a protein sequence [36]. A similar prediction analysis in Trypanosomatids using TargetP for mitochondrial location prediction has been done elsewhere [37]. For transport of a protein to the glycosomes, presence of a peroxisomal targeting sequence (PTS-1 or PTS-2) is required [38–40]. ScanProsite [41] was used for predicting the presence of a PTS signal. PTS-1 can be detected by locating the PROSITE signature PS00342 within a sequence, which determines the requirement of presence of a particular tripeptide at the C-terminal of the protein sequence [39]. PTS-2 was detected by finding a variable length PROSITE pattern <M-x(0,20)-[RK]-[LVI]-x(5)-[HKQR]-[LAIVFY] at the N-terminal of the protein sequence [39].

Almost all glycosomal proteins have isoelectric point (pI) in the range of 8.8–10.2 [20, 42, 43]. In order to further confirm the glycosomal localization of proteins, isoelectric point for every protein was predicted *in silico* using the ProtParam Tool [44]. For example, there are two gene copies encoding the asparaginase protein (UniProt IDs: A4HWE1, A4IDM2). The predicted pI of A4IDM2 was 9, whereas for A4HWE1 the predicted pI was 5.7. Based on this information, the two proteins, even though similar, were assigned glycosomal and cytosolic locations respectively.

Based on all the above mentioned analysis, a confidence score was evaluated for the location of every reaction. The confidence score is designed in such a way that prediction of an enzyme’s location when supported by literary evidence is given more importance and the assignment of location solely based on sequence analysis is considered to be less significant (see S1 Table and Section A in S1 Text). The reactions for which localization confidence score was 2 or less, an additional analysis was performed for confirmation (see Section B in S1 Text and S2 Table). The top scoring sequence homologues (E-value<1e-10) other than from the genus *Leishmania*, obtained through sequence search were also scanned for the presence of mitochondrial targeting peptide or peroxisomal targeting signal. If these selected homologues also displayed the presence of targeting signals, then a higher confidence was given to the probable location assigned. If not, then the assignment of function was made purely based on the results acquired from sequence-based prediction of subcellular location with low confidence. For certain reactions, the subcellular location was not clearly identified through experiments, and is rationally assumed to occur in a particular subcellular location. One such reaction is glycosomal triose phosphate isomerase (TPIg), which is assumed from experiments to occur in the glycosome although the sequence-based subcellular location analysis does not indicate presence of any subcellular signals. For such reactions, a score of 2 was assigned so as to account for the uncertainty of its subcellular location through experiments. Thus, TPIg was assigned to the glycosome with a score of 2 and then included in the network.

Apart from this, certain transport reactions could be associated with their corresponding genes with respect to the information available from the TransportDB database [45]. From TransportDB, information about membrane transporters within *Trypanosoma* and *Leishmania* and their associated substrates was collated. Protein sequences with considerable sequence similarity to the above given transporter protein sequences were then associated with transport of the particular substrates considered in our model (see S1 Table). Similarly, intracellular transport reactions between the glycosome-> cytosol and cytosol-> mitochondria reported through literature have also been included in iAS142 [18, 19, 46, 47]. The intracellular transports include the transports of inorganic molecules like H_2_O, H+, etc., transports of fermentation products like lactate, succinate, pyruvate, CO_2_, other C4 dicarboxylic acids like fumarate, malate, etc., transport of amino acids and shuttle pathways between different compartments. The glycosomal membranes of the trypanosomes are thought to be relatively impermeable to adenine nucleotides like ATP/ADP and NAD/NADH, thus maintaining their ratio inside the glycosome [48]. Hence, their transport across the glycosomal membranes was not considered. Also, exchange and transport of acetyl coA was assumed in the model to compensate for absence in uptake of fatty acids in the model. Similarly, extracellular transports and exchange reactions were added to the model with respect to the presence of organic and inorganic sources that are experimentally known to occur in the environment of the parasite [18, 19, 46, 47].

### Naming convention

According to the previously established convention of naming *in silico* constraint based models [49], the computational model was named ‘iAS142’. Model names begin with ‘i’ indicating *in silico*, followed by the first author’s first and last initials (‘AS’) followed by the number of genes that are part of the model (142).

### Flux Balance Analysis (FBA)

The list of reactions along with the metabolites was transformed into a stoichiometric matrix *S*, a mathematical representation of the reconstructed network. The *S* matrix comprises of *m* reactions and *n* metabolites (dimensions of *S* are *m* x *n*). Each element in *S*_*ij*_ represents the stoichiometric coefficient of metabolite *i* in reaction *j*. The coefficients are positive if the concerned metabolite *i* is a product in reaction *j* and negative if a reactant. The metabolic iAS142 model comprises of 237 reactions and 231 metabolites highly interconnected between 5 distinct model compartments. The stoichiometric matrix *S* for this system is of size 231 **x** 237. The vector *v* has 237 fluxes, including 27 exchange fluxes. Note that each reaction flux in the flux vector is constrained between bounds *a* and *b*. All reversible reactions considered in the model were given bounds as *a* = -1000 and *b* = 1000, the default considered in rBioNet toolbox [26]. The bounds for the irreversible reactions considered in the model were *a* = 0 and *b* = 1000. For metabolite exchange reactions, exchange specifically considering release of a particular metabolite from the cell were bounded between *a* = 0 and *b* = 1000, whereas exchanges considering uptake of a metabolite were bounded between *a* = -1000 and *b* = 0. After applying the required constraints, the model was analyzed using Flux Balance Analysis (FBA) [14, 50].

### Objective function

The objective function used in previous studies was constructed using biochemical data of cellular composition, available in the literature for various organisms [15, 17, 51]. Previous constraint-based models on Trypanosomatids have used biochemical data from distant organisms like *Bacillus* and *Tetrahymena* respectively, to construct the objective function [15, 17]. In comparison to these studies, we have attempted to formulate a novel biomass objective function that represents a metabolic demand reaction, a drain to specific metabolites present in the model. This metabolic demand reaction (coefficient of the metabolites in the biomass objective function reaction) was derived from the 13C isotopic enrichment data measured in *L*. *mexicana* developmental stages, where glucose in the medium was labeled with 13C isotope [19]. See Section A in S1 Text for details behind formulation of the biomass objective function.

The cultivation of the different developmental stages in *Leishmania* is performed in a completely defined medium (CDM) which is majorly composed of glucose when compared to the other carbon sources [52]. Glucose has also been shown to be utilized in both the life stages of *Leishmania* [53]. Additionally, most enzymes of the glucose catabolism have been shown to be essential for either survival or virulence of trypanosomatids [20, 22, 53–55]. Hence, we assumed that, as glucose largely affects the metabolism of *Leishmania* as compared to other carbon sources, the biomass objective function based on the 13C isotopic enrichment data attained from a CDM containing 13C labeled glucose, would be as close to the actual metabolic demand reaction of *L*. *mexicana*. As a proof of concept, this derived biomass objective function can possibly be applied to *L*. *infantum*, considering it to be an evolutionarily close relative of *L*. *mexicana*. It is important to note here that, none of the previous metabolic reconstructions have considered radio-labeled isotopic enrichment data for deriving the objective function. Further, it can be observed that the biomass reaction accounts for precursor metabolites of different pathways related to energy and non-essential amino acid metabolism in *Leishmania*. Further, other energy and amino acid metabolic reconstruction studies of Trypanosomatids also considers precursor metabolites in their biomass reaction [15]. Also, ATP drain in the network, considered together with the biomass equation by previous models [15, 17, 51] was considered separately in iAS142. The terms for glutamate, alanine, and aspartate in the biomass reaction represent a drain for non-essential amino acids that can be primarily synthesized through the TCA cycle. The synthesis of non-essential amino acids through TCA requires a redox balance to be maintained within the mitochondrion through oxidative phosphorylation which drives cellular ATP production. The ATP produced is then drained through the cytosolic ATP maintenance that recycles ADP and Pi for ATP production. The considered biomass thus, accounts for maintenance of cellular energy requirements also, as it is coupled with the ATP maintenance in our model through redox balance. To compare our newly formulated biomass objective function with an existing biomass objective function (previously used in the iSR215 energy metabolism model in *Trypanosoma cruzi* [15]) we simulated our network under specific conditions of glucose and amino acid uptake.

### Model simulations and analysis

Model simulations were performed by changing the bounds of different reactions and validating them with existing biological observations. Exchange for glucose was constrained such that there will be uptake of glucose. Exchanges of non-essential amino acids were selectively opened along with glucose to understand their fate. The exchanges of essential amino acids have been constrained to zero, to ensure that the model simulations are dependent only on glucose as an essential substrate. Also, exchange of overflow metabolites were constrained to be excreted outside the cell. The default model simulations correspond to the situation where glucose is abundant and the sole carbon source in the environment. Flux balance analysis of the network was performed using the COBRA toolbox [56]. For sketching the actual map figure, the map coordinate file for generating the iAS142 metabolic map was manually prepared in the format accepted by the COBRA toolbox.

### Reaction knockouts

To identify the reactions essential for parasite growth and to simulate the effect of the single reaction deletions, the upper and lower bounds of all the reactions were constrained to be zero (*a*_*i*_ = *b*_*i*_ = 0), sequentially one reaction at a time, and FBA was performed subsequent to each reaction knockout in a medium containing only glucose. Further, to perform double reaction knockouts, the bounds of different reaction pairs were constrained to be zero and FBA was performed subsequent to each combination knockout keeping glucose as the sole carbon source. In each of these knockout simulations, the transport fluxes of glucose and oxygen were fixed to their optimal flux value attained from the wild type simulation (the default FBA run devoid of reaction knockouts). Glucose is present as its anomers—alpha-D-glucose (Aglc-D) and beta-D-glucose (Bglc-D) in the model which interconvert by the reaction catalyzed by aldose-1-epimerase. Accordingly, transports for alpha-D-glucose and beta-D-glucose have also been considered separately in the model. Therefore, in order to avoid degeneracy of glucose uptake from either of these transports, one of them was closed during reaction knockout study. For both the single and double reaction knockout studies, a reaction proved to be essential (lethal phenotype) when biomass reaction flux became zero after applying the aforementioned constraints; else it was deemed to be non-essential (non-lethal phenotype).

#### Validation through reaction knockouts

Results from diverse experimental techniques that involved targeted gene deletion, RNAi mediated repression, and inhibition of reactions by small molecule analogues were considered for validation purpose. For validating the reaction knockout results, only growth phenotypes obtained from knockout studies carried out in *Leishmania* species were used. As literature proof for only single reaction deletions was available for *Leishmania*, only single reaction knockout studies were used for validation purpose.

#### Comparison of reaction knockout phenotypes with other Trypanosomatids

In order to confirm the model to be *Leishmania* specific, we compared the reaction knockout growth phenotypes predicted from our model with phenotype information gathered from knockout studies carried out in different Trypanosomatids. This comparison was explicitly performed for delineating the differences between the model-predicted growth phenotypes and the observations from experimental knockout studies carried out in *Trypanosoma* species; and not for validating our model.

### Validation through experimental data

For further validation of the model, we sought to look for the secretion of metabolites out of the network. These metabolites are known to be secreted by *Leishmania*, and numerous studies have been carried out to understand their importance in the parasite’s metabolism [6, 57, 58]. Metabolites like acetate, succinate, lactate and pyruvate are the end products of fermentation, which are mainly secreted under hypoxic conditions. Hence, variation in uptake flux of extracellular oxygen from its optimal value may lead to the secretion of these aforementioned metabolites. Accordingly, extracellular transport of oxygen was decremented from its near optimal value to 0 and FBA was performed for each of these values. The flux through each of these secretion products’ transport was monitored and plotted against its corresponding oxygen uptake flux value.

#### Comparing *L*. *infantum* iAS142 with other Trypanosomatid reconstructions

Previously, among the Trypanosomatids, the metabolic networks of *L*. *major* (iAC560 model) and *T*. *cruzi* (iSR215 model) have been reconstructed and analyzed using flux balance analysis [15, 17]. As the energy metabolic network of *L*. *infantum* iAS142 model has been curated *de novo* for the *L*. *infantum* genome with respect to updated information obtained from literature, it became important to identify the significant differences with respect to energy metabolism between the three models. The differences in reaction subcellular locations and metabolites used in the three models were compared and enlisted. These differences can help to understand the differences between behaviors of the metabolic networks.

Further, to assess the effects of these model differences on network behavior, reactions representing the glucose and non-essential amino acid network of the *L*. *major* iAC560 were extracted separately. The bounds of the individual reactions were also fixed to the same values in both the models. FBA was performed using the iAS142 biomass function; both for the iAS142 metabolic network and for the corresponding subset of reactions from the *L*. *major* iAC560 genome scale metabolic network. To quantify the differences, fluxes through the exchange reactions and biomass reactions from each of the models were monitored. To understand the degree of association between the *L*. *major* iAC560 and *L*. *infantum* iAS142 flux profiles, the non-parametric Spearman correlation was used. Students’ t-test was used to compare the magnitude of fluxes of reactions related to glycosomal lactate fermentation between the two models [59].

#### Effect of amino acids with glucose on model reaction fluxes

The stoichiometry of amino acid uptakes in the model is from cytoplasm to extracellular space. Hence to impose constraints such that the amino acids are only taken up by the network, the upper bound of the exchange flux of aspartate, glutamate, proline and alanine amino acids was fixed to 0 (zero) and the lower bound to -1000. FBA was performed under these constraints for measuring each amino acid uptake flux value. The flux profiles were saved and the important reactions shown in the heatmap were grouped according to the pathway they belong. In order to understand the effect of all amino acid uptakes when coupled with glucose uptake, the upper bounds of all four of these amino acid exchange fluxes were simultaneously fixed to 0 (zero) and the lower bound to -1000. In all the simulations, exchanges of essential amino acids have been kept zero as pathways involved in catabolizing them have not been considered in the iAS142 network.

#### Stage specific energy metabolism of *Leishmania infantum*

*L*. *mexicana* developmental stages were previously shown to co-utilize glucose and a few, selected non-essential amino acids like glutamate, aspartate, alanine and proline, when cultured in a CDM containing a range of carbon sources [18, 19]. Hence, to simulate promastigote metabolism, iAS142 was constrained for intake of glucose along with non-essential amino acids like aspartate, glutamate, alanine and proline and FBA was performed to maximize the iAS142 biomass. Proteomic analysis in *L*. *infantum* identified preferential expression of hexokinase, ATP-dependent phosphofructokinase/6-phospho-1-fructokinase, glycosomal glyceraldehyde-3-phosphate dehydrogenase, and PAS-domain containing phosphoglycerate kinase in promastigotes as compared to amastigotes [10]. Thus, for simulating the amastigote stage, at least with respect to the energy metabolism, the upper and lower bounds of all the aforementioned reactions was fixed to 10% of the promastigote flux value. A similar strategy to elicit the stage specific metabolism in *L*. *major* was used elsewhere [17]. FBA was performed for this constrained scenario and the flux profile was monitored. To understand the degree of association between the model promastigote and amastigote flux profiles, the Spearman correlation was used [59].

#### Simulating therapeutic intervention

To simulate intervention by chemotherapeutics in the amastigote stage, we varied the optimal flux values of the previously determined targets in the model-presumed amastigote stage and attempt to monitor the corresponding growth and ATP synthesis rates. For this simulation, the upper/lower bound of each target (with respect to its default bounds) was evenly varied from its optimal flux value observed in the amastigote stage presumed in the model up to zero. For every change in the bounds of the target reactions, a separate FBA simulation was performed by maintaining glucose as the only carbon source, to capture the steady state growth and ATP synthesis.

## Results

### Model Properties

The iAS142 metabolic network consists of 237 total reactions spanning 5 different subcellular compartments. Out of the 237, around 109 metabolic reactions, 99 transport, 27 exchange reactions, a metabolic demand reaction (biomass), and an ATP maintenance reaction constitute the iAS142 network. A total of 115 reactions could be associated with 142 genes from the *L*.*infantum* genome (Table 1). Notably, the metabolic demand reaction considered in this study is different from the biomass drain reactions employed by previous similar studies (as it was formulated using isotopic enrichment data acquired from 13C isotope resolved metabolomics) [19].

**Table 1:** Properties of the iAS142 constraint-based model.

Property	Count
Genes	142
Reactions	237
Gene associated (intracellular)	108
Gene associated (transport)	7
Non-Gene associated (intracellular)	1
Non-Gene associated (transport)	92
Exchange	27
Demand (Metabolic)	1
ATP maintenance reaction	1
Metabolites	231
Compartments	5
Literature References (for reconstruction)	86
Databases Consulted (for reconstruction)	10

**Fig 2:**
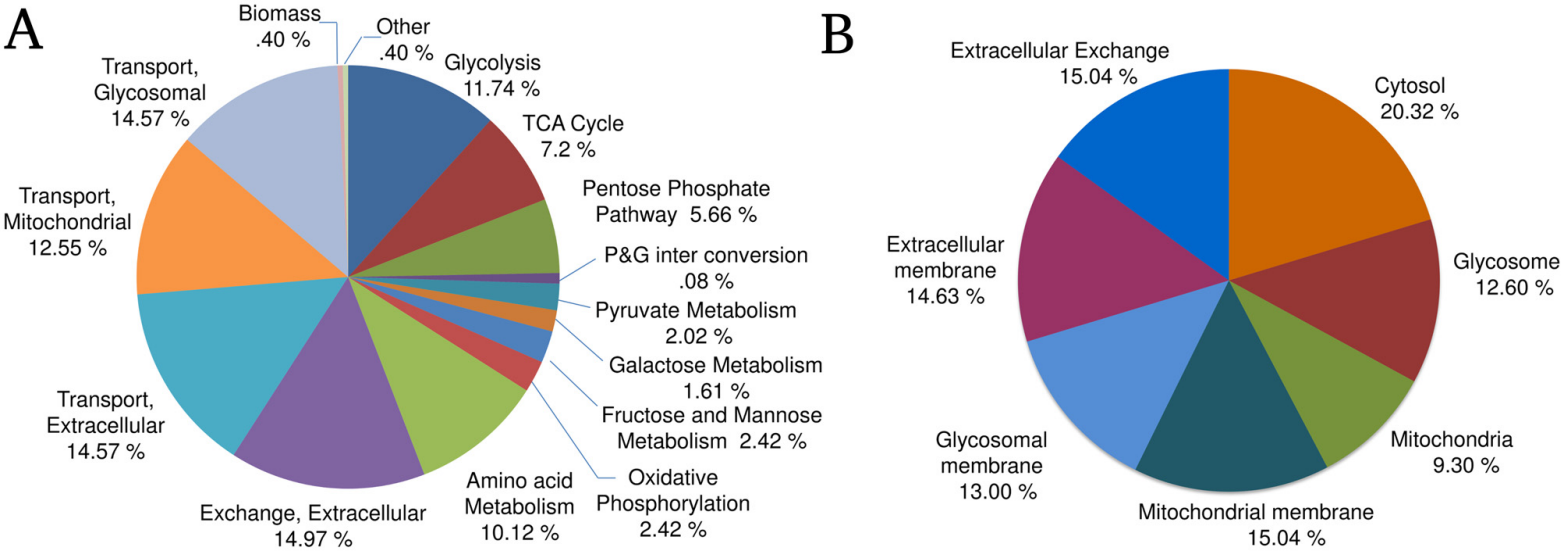
A) Pie chart showing the pathways that comprise the iAS142 model B) Pie chart showing the percentage of model reactions belonging to different subcellular locations.

Fig 2A depicts a pie chart that classifies reactions in the model according to the pathways in which they fall. Glycolysis, Citric acid cycle and Pentose Phosphate pathway constitute approximately 25% of the total reactions in the model. Amino acid metabolic pathways also represent a major portion of around 10% of the total, demonstrating the importance of integrating them with the carbohydrate metabolic pathways for studying energy metabolism. The metabolic network presented in this study does not encompass the full genome of *L*. *infantum*, by virtue of which the percentage of the reactions occurring in the cytosol, mitochondria and the cytosol appear to be nearly equal (Fig 2B). Also, around 42% of the total reactions are present in membranous compartments representing the intra-cellular and extracellular transport reactions for various metabolites.

**Fig 3:**
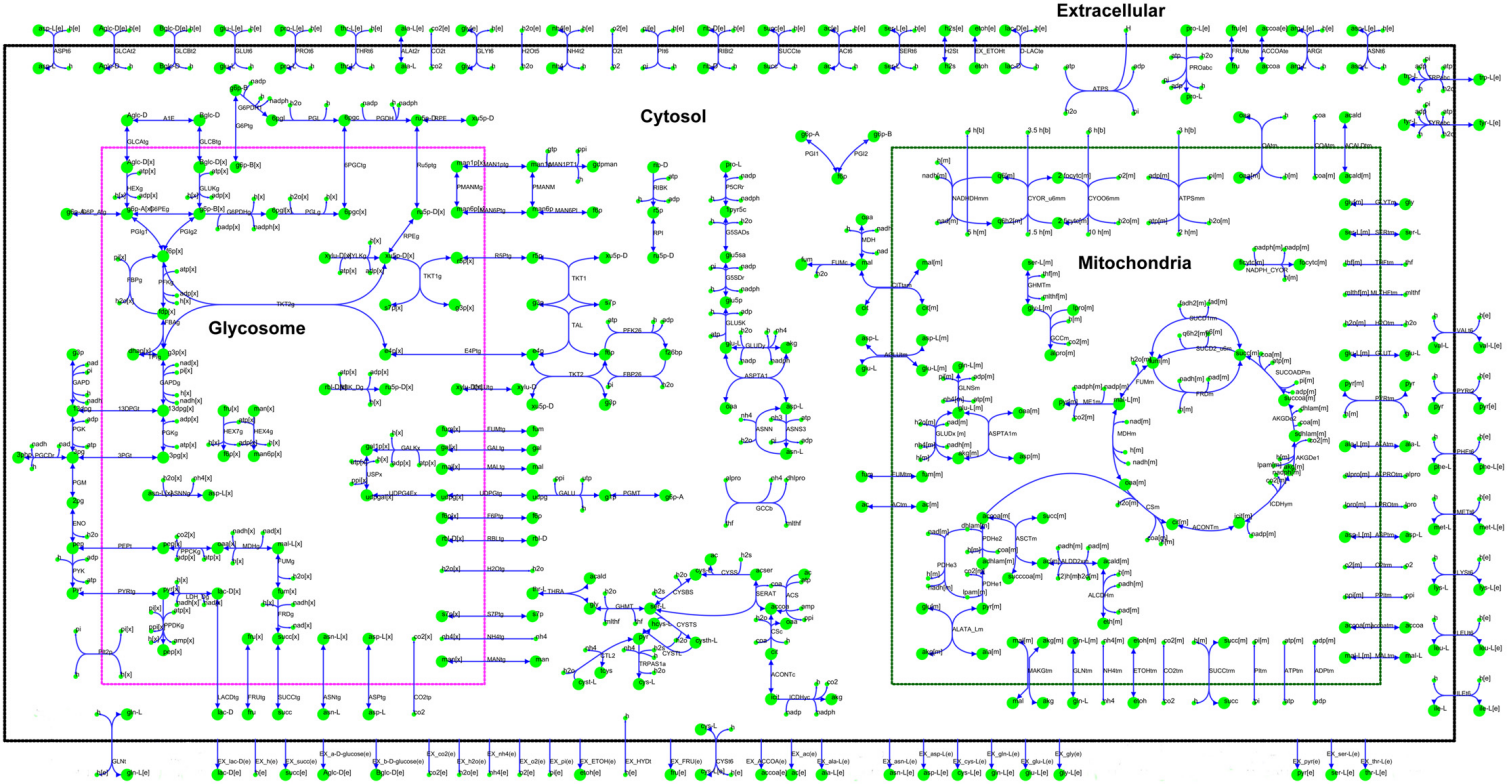
The map of the iAS142 metabolic network—The iAS142 network comprises of 237 reactions that occur in 5 major model compartments: 4 cellular compartments—the glycosome, cytoplasm, the mitochondrion, the mitochondrial inter-membrane space and 1 extracellular compartment as shown in the Fig. The mitochondrial inter-membrane space though included in the model, is not explicitly shown in the Fig. The reactions of oxidative phosphorylation occur from the mitochondrial compartment (m) to the mitochondrial inter-membrane space (mm) and vice versa. The reactions occurring along the borders of the compartments are transport reactions. The metabolite exchanges have been shown at the bottom of the Fig.

With respect to the pathways considered in the iAS142 network, glycosomes are linked primarily with glycolysis, pentose phosphate pathway and fermentative pathways of C4 dicarboxylic acids; while, mitochondria accounts for TCA Cycle, oxidative phosphorylation, pyruvate metabolism and few amino acid metabolic pathways (Fig 3).

### Improving annotation and predicting the localization of enzymes

The iAS142 metabolic network was reconstructed keeping in mind the association between the gene, protein and their associated reactions. There were also a number of non-gene associated reactions primarily governing transports of metabolites within the cell. Primary sequence analysis for every protein enlisted in S1 Table was performed in order to affirm the function of that particular protein. A putative glucose-6-phosphate epimerase (UniProt ID—A4IAA4) previously annotated as aldose-1-epimerase, was re-annotated for its substrate level function by identifying conserved active site residues specific for recognizing the glucose-6-phosphate substrate through sequence comparison with the protein sequence of a recently annotated glucose-6-phosphate epimerase PDB structure (PDB ID = 2CIR). Structural analysis was performed to identify these residues that interact with the phosphate moiety of glucose-6-phosphate in the 2CIR structure (See Methods section). The associated information of the same is reported in S1 Table.

As *Leishmania infantum* is a unicellular eukaryote, its cell is composed of many intracellular compartments. As mentioned before, we have included 5 different model compartments: four subcellular and an extracellular for transport and exchange of metabolites. Every reaction in the network was assigned to its respective compartment both on the basis of availability of literature evidence and also through sequence based prediction of subcellular localization. For around 54 reactions, we could obtain the information about the subcellular localization through previously published literature as well as predict location through sequence analysis (see S1 Table). These reactions can be identified by a high confidence score (= 5) in the model. For 22 reactions, subcellular location was annotated solely through sequence-based prediction of cellular localization signals (see Section B in S1 Text). These reactions can be identified by a low confidence score (< 5) in the model. The sequences of proteins corresponding to the reactions were used for prediction of subcellular localization and checked for presence of a cellular localization signal (See Methods section).

Before assigning a specific compartment to a particular reaction, further confirmation of subcellular localizations was performed by finding sequence homologues with high sequence identity to our protein of interest and probing them for the presence of the mitochondrial and peroxisomal signal sequences (See Methods section for details). Presence of these targeting signals in the close homologues could further increase the confidence about probable location of the protein. Precisely, 11 enzymes were assigned to their appropriate subcellular locations with respect to localization signals detected in its closest homologues (see S2 Table). If the subcellular location of a reaction could not be confirmed by this technique, the assignment of location was made purely based on the results obtained from sequence-based detection of subcellular location but with low confidence. Accordingly, 7 enzymes were allocated their locations purely on the basis of sequence-based detection but with low confidence (Table SB of Section B in S1 Text).

We further compared the *L*. *infantum* and *L*. *major* enzymes of energy metabolism through *in-silico* sequence-based predictions for differences in subcellular locations. We could identify that the subcellular locations predicted for both *L*. *infantum* and *L*. *major* enzymes were the same; the only major difference being the predicted subcellular location of glutamine synthetase. In *L*. *infantum*, glutamine synthetase was predicted to be localized in the mitochondria, whereas it was predicted to be localized in the cytoplasm in *L*. *major* due to probable absence of a mitochondrial targeting peptide signal within its sequence. Only the *L*. *infantum* and *L*. *major* protein sequences related to energy metabolism were considered for this comparison. The comparison between the reaction subcellular locations as annotated in the iAS142 and iAC560 reconstructions have been discussed later. Also, a thorough literature search indicates the requirement of a major experimental study that can further identify differences in subcellular locations of enzymes between *L*. *infantum* and *L*. *major*. One such study identified the presence of a cytoplasmic glyceraldehyde-3-phosphate dehydrogenase through experiments to be specific to *L*. *infantum* and absent in *L*. *major* [60]. Hence, glyceraldehyde-3-phosphate dehydrogenase was kept to be cytoplasmic in the *L*. *infantum* iAS142 model.

### Reaction knockout analysis and predictions

Essential genes required for survival of an organism can be found by performing *in-silico* deletion of genes in constraint-based models and investigating its consequences on growth and proliferation of the organism. Deletion of any single gene might predict that it is non-essential even though the reaction on the whole, might be essential for parasite growth. Every reaction in our model essentially represents a set of common genes associated with respective isozymes catalyzing the corresponding reaction. Thus, instead of gene deletions, an *in-silico* reaction knockout study would give a better perspective of checkpoints in the pathway that could be controlled by a chemotherapeutic and may lead to reduction in growth of the parasite (See Methods section for details). We performed both single and double reaction knockouts to predict the subset of reactions that are essential for parasite growth (Table 2 and S3 Table).

**Table 2:** Reaction lethality predictions.

Reaction Deletion	Lethal	Trivial lethal	Non-trivial lethal	Non-lethal	Total cases
Single	61	NA	61	153	214
Double	10884	10829	55	33636	44520

Trivial lethal—atleast one of the reactions in the double deletion pair is lethal in a single reaction deletion

Non-trivial lethal, single—reaction involved in the single deletion is lethal

Non-trivial lethal, double—reaction pair involved in the double deletion is lethal

NA—Not applicable.

#### Validation of reaction knockouts and predictions

Reactions essential for parasite growth could be predicted by performing single reaction knockouts in the *L*. *infantum* iAS142 model (see S3 Table). 61 reactions out of the total 142 reactions were predicted to be lethal (Table 2). Previously, in the *L*. *major* iAC560 model study, the growth phenotype information from knockout studies in closely related *Trypanosoma* species has been considered for the validation of reaction knockouts in their model [17]. But, both bloodstream and insect forms of *Trypanosoma* live in an environment that is entirely different from the *Leishmania* species. Hence, it would be inappropriate to explicitly validate the knockouts of *Leishmania* using *Trypanosoma* data. Experimentally determined growth phenotype information for only a few knockouts (7 knockout phenotypes), are explicitly available for any *Leishmania* species (Table 3). Although *Leishmania* specific knockout phenotypic information available to validate this analysis was limited [61–66], the predicted lethality for model reaction knockouts exactly matched with the previously known growth phenotype information obtained for the genes from gene knockout studies carried out in *Leishmania* species (Table 3). The model knockouts were also compared to knockouts predicted from the *L*. *major* iAC560 model as well (see Section C & Table SC in S1 Text). The comparison suggests that the *L*. *infantum* iAS142 model performs better in predicting actual growth phenotype scenarios. Even though the amount of *Leishmania* specific information required for making a firm statement about the model validity was insufficient, regardless to say, the absolute accuracy of the model in predicting the few known *Leishmania* specific growth phenotypes could suggest the ability of the model in making biologically agreeable predictions.

**Table 3:** Reaction knockout validations of the iAS142 metabolic network.

Model reaction constrained	Experimental Target	Model prediction	Experimental finding	Reference (for experiment)	Organism	Predicted wild type growth (%)
ACONTm	Aconitase	lethal	lethal	[19]	*L*. *mexicana*	0%
ATPSmm	ATP synthase (Mitochondrial membrane)	lethal	lethal	[61]	*L*. *donovani*	0%
CYOO6mm	Cytochrome c oxidase (Mitochondrial membrane)	lethal	lethal	[62]	*L*. *donovani*	0%
FBPg	Glycosomal fructose 1,6 bisphosphatase	nonlethal	nonlethal	[63]	*L*. *major*	99.99%
MAN6PI	Phosphomannose isomerase	nonlethal	nonlethal	[64]	*L*. *mexicana*	99.99%
PMANM	Phosphomannomutase	nonlethal	nonlethal	[65]	*L*. *mexicana*	99.99%
USPx	UDP-sugar pyrophosphorylase	nonlethal	nonlethal	[66]	*L*. *major*	99.99%

Biologically, certain reaction knockout predictions from the model may have very important consequences from the perspective of the parasite energy metabolism and hence, might govern a lethal phenotype. Succinate fermentation takes place in the glycosomes and involves formation of succinate from phosphoenolpyruvate by virtue of four sequential reactions—PPCKg catalyzed by phosphoenol pyruvate carboxykinase, MDHg catalyzed by malate dehydrogenase, FUMg catalyzed by fumarate hydratase, and FRDg catalyzed by fumarate reductase. Through these reactions, NAD molecules consumed by the enzymes of upper glycolytic pathway are regenerated [6, 18]. Hence, these reactions occurring in the glycosome govern a lethal phenotype. Interestingly, NAD could also be regenerated through lactate dehydrogenase reaction which also takes place in the glycosome. But the inability of the model to produce biomass in the absence of succinate production, immediately points towards the presence of succinate in the metabolic demand reaction itself. This result shows the importance of choice of biomass function in a constraint based model and analysis. For analyzing the variation in predictions due to choice of biomass function, we have studied two different biomass equations, as further illustrated later in this section.

NADPH cytochrome oxidoreductase (NADPH_CYOR) a reaction that was not considered in previous Trypanosomatid core energy metabolism reconstructions [15] is required to maintain the NADPH redox balance in the mitochondrion by regeneration of NADP from NADPH, thus, rendering it to be essential. Similarly, NADPH-dependent glutamate dehydrogenase (GLUDy) reaction is the only reaction considered in the model that helps in regeneration of NADP from NADPH in the cytosol. Therefore, it is predicted to be indispensible. Also, on the other hand, cystathione beta lyase (TRPAS1a) and glucose-6-phosphate dehydrogenase (G6PDH1) reduce NADP back to NADPH, making them crucial. Ammonium (NH_4_) is used as a substrate for GLUDy and TRPAS1a reactions. Without ammonium transport, the GLUDy and TRPAS1a reactions would be affected creating an imbalance in NADP-NADPH ratios which would be deleterious to the cell. Hence, ammonium transport also governs a lethal phenotype. Further, mitochondrial membrane ATP synthase (ATPSmm) that is responsible for ATP production also governs a lethal phenotype suggestive of its coupling with growth (although not included in the iAS142 biomass reaction) whereas the cytosolic ATPase reaction (ATPS) that represents a continuous drain of ATP is not dependent on growth maximization and hence, is not predicted to be lethal.

In addition to single reaction deletions, we also performed double reaction deletions of all possible paired combinations of reactions considered in the model (Table 2). A total of 10884 lethal combinations could be identified; out of which there were 10829 “trivial” cases, i.e. at least one of the reactions in the pair was lethal in the single reaction knockout study. There were 55 “non-trivial” lethal combinations (see Table 2 and S3 Table), i.e. both genes involved were not lethal individually but were lethal in a combination. These predicted novel lethal reaction combinations could be possible combinatorial therapeutic targets, each combination providing an experimentally testable hypothesis.

#### Comparison of reaction knockouts with knockouts in related Trypanosomatids

The predicted reaction knockout phenotypes were further compared with related Trypanosomatids. This comparison was not performed to validate the model, but to demonstrate differences in closely related organisms residing in different environments. Single and double reaction knockouts performed in the iAS142 model led to the identification of reactions essential for parasite growth. As data for double deletion studies in Trypanosomatids were relatively scarce for enzymes considered in the model, only single reaction knockout predictions were used for this comparison. For comparison of the predicted growth phenotypes with *Trypanosoma* species, we restricted ourselves to the insect-stage procyclic *T*. *brucei* phenotype information, as the promastigote and *T*. *brucei* insect stages exist in fly midgut. Comparison with every known growth phenotype in the insect stage *Trypanosoma* species further revealed that few growth phenotypes in *Trypanosoma* show substantial difference with the iAS142 model predictions (see Section D & Table SD in S1 Text). For instance, procyclic *T*. *brucei* have been shown to avoid Krebs cycle for energy generation [16, 67]. Hence, mitochondrial aconitase deletion in *T*. *brucei* does not demonstrate a lethal phenotype. On the contrary, *Leishmania* species are shown to display an active Krebs cycle in both the promastigote and amastigote stages being a necessity for its growth [18, 19, 68]. Probably, this difference acknowledges the constitutive and essential requirement of mitochondrial aconitase, specifically in *Leishmania* species as rightfully predicted from the model. Similarly, UDP-glucose/galactose 4-epimerase was proven to be essential in both the stages of *T*. *brucei* [69]. In *Leishmania* species, it still needs to be experimentally proven if this enzyme governs a lethal growth phenotype. But, the iAS142 model reaction knockout predicts UDP-glucose/galactose 4-epimerase to be non-lethal confirming that this model prediction does not agree to the phenotype observed in *Trypanosoma* species. Other comparisons of iAS142 model predictions with phenotype information of *Trypanosoma* [70–72] are further reported in Section D of S1 Text.

### Simulation of the secretion of overflow metabolites

**Fig 4:**
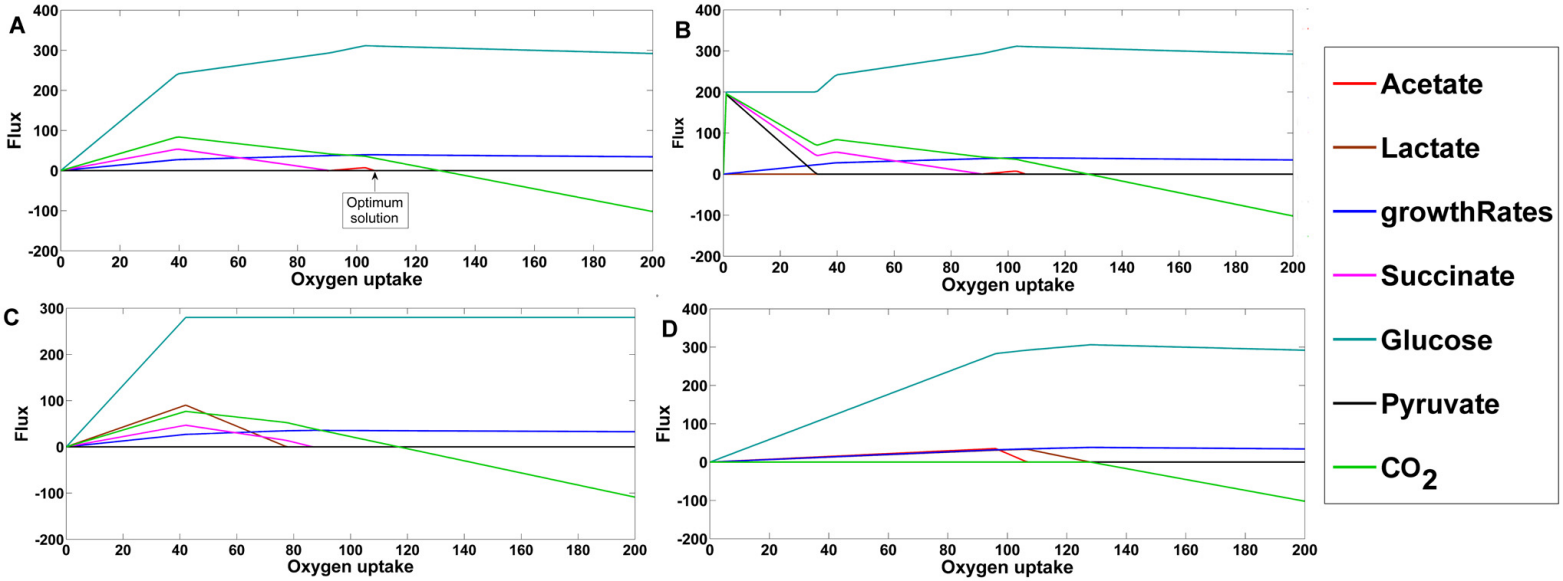
Robustness analysis with respect to oxygen uptake to simulate overflow metabolite secretion. A) Secretion of different overflow metabolites and glucose uptake with variation of oxygen uptake B) Secretion of different overflow metabolites and glucose uptake with variation of oxygen uptake by fixing lower bound of glucose uptake to a flux value of 200 C) Secretion of different overflow metabolites with variation of oxygen uptake by fixing upper and lower bounds of glucose uptake to a flux value of 280. The constraints for glucose uptake were changed to its default values at oxygen uptake rate < = 40 as the simulation gave no solution. D) Secretion of different overflow metabolites with variation of oxygen uptake and fixing lower bound of CO_2_ to a value of -1000 and upper bound to 0.

Overflow metabolites are those metabolites which are secreted from the cell due to high uptake of glucose or other carbon sources. *Leishmania* is known to exhibit an overflow metabolism under high glucose or low oxygen conditions, during which it secretes substantial amounts of overflow metabolites like succinate, acetate, pyruvate, CO_2_ and small amounts of lactate [6, 57, 58]. As a second level of model validation, we sought to observe the secretion of these metabolites in our model by variation of oxygen uptake. At the optimum solution of oxygen uptake, clearly there was no secretion of overflow metabolites (Fig 4A). At the optimum solution of oxygen uptake, glucose is catabolized completely for production of succinate which enters into the TCA cycle (instead of being secreted out) so as to maximize biomass and thus, ATP synthesis. This result indicates that the parasite demonstrates a glucose-dependent metabolism for its survival, where it is well adapted to maximize its growth and ATP synthesis by complete utilization of glucose and oxygen from the environment. But, as the flux value of oxygen uptake decreases, acetate immediately starts to be secreted; followed by succinate, on further decrement of oxygen uptake (Fig 4A). The glucose uptake rate decreases in a piece wise linear fashion with respect to the decrease in oxygen uptake rate. Albeit, fixing the lower bound of glucose uptake to an arbitrary value of 200 consequently displayed the secretion of pyruvate and succinate (Fig 4B). The bounds of glucose uptake were fixed to 200, as pyruvate secretion is observed for glucose uptake between 190 and 210 under constant low oxygen uptake. Pyruvate is secreted due to the increased flux through pyruvate kinase and succinate fermentation enzymes that helps maintaining the ATP/ADP ratio in glycosome and cytosol under highly anaerobic conditions. Similarly, constraining both the upper and lower bounds of glucose uptake rate to 280 led to the secretion of lactate (Fig 4C). This is because, glucose uptake between 0 and 280 under constant low oxygen uptake leads to lactic acid secretion. Under this constraint, glucose is distributed between the preferred succinate fermentation pathway and the D-lactate dehydrogenase reaction thereby leading to secretion of succinate and lactate. Under anaerobic constraints, high uptake of glucose leads to its conversion into succinate and lactate fermentation. The constraint on glucose uptake rate was removed when oxygen uptake rate decreased to a flux value of 40, as there is no solution of the simulation in that range, indicating infeasibility of such constraints in real situation (Fig 4C).

The stoichiometry of CO_2_ transport in iAS142 is from extracellular space to cytoplasm. Thus a positive flux value through its transport reaction means that CO_2_ is transported into the cell from extracellular environment. During simulations, CO_2_ transport is positive for flux values through oxygen uptake just below 130, indicating its intake into the network. To understand the importance of CO_2_ uptake, we restricted the flux through it in the negative direction and continued the simulations by varying the flux through oxygen uptake (Fig 4D). From the Fig it can be observed that secretion of succinate halted spontaneously and lactate and acetate secretion commenced profoundly. These results are allusive of the interplay between the glucose and oxygen uptakes, which may influence the final metabolites that are to be secreted. Moreover, CO_2_ may be essential for the secretion of succinate during overflow metabolism.

### Comparison of *L*. *infantum* iAS142 with other Trypanosomatid reconstructions

**Fig 5:**
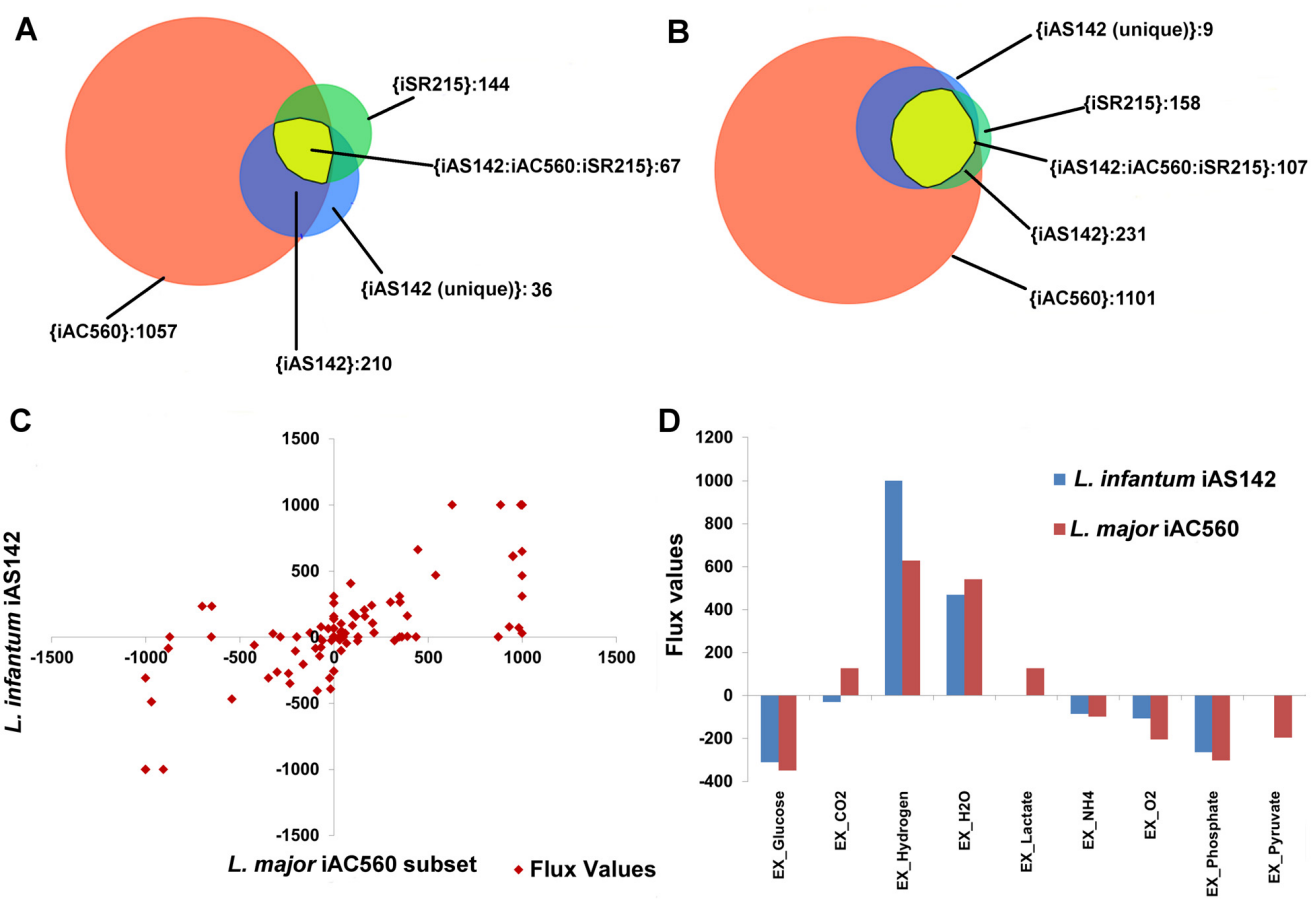
Comparison of the *L*. *infantum* iAS142 network with other Trypanosomatid reconstructions. A.) Venn diagram showing the comparison between intracellular reactions (exchanges excluded) considered in the *L*. *infantum* iAS142, *L*. *major* iAC560 and *T*. *cruzi* iSR215 reconstructions. B.) Venn diagram showing the comparison between the metabolites considered in the *L*. *infantum* iAS142, *L*. *major* iAC560 models, and *T*. *cruzi* iSR215 reconstructions [the brackets ‘{}’ represent the set of elements constituting the particular area in the Venn Diagram]. C) Scatter plot showing comparison of the *L*. *infantum* iAS142 model with energy metabolism subset of *L*. *major* iAC560 model. D) Comparison of secretion of overflow metabolites between the *L*. *infantum* iAS142 and the energy metabolism subset of *L*. *major* iAC560 model—bar graph representing exchange fluxes from both the models.

Although, the real energy metabolism between *L*. *infantum* and other Trypanosomatids might be conserved, there are numerous differences with respect to the energy metabolic networks reconstructed for these species. For this purpose, the iAS142 reconstruction has been compared with the *L*. *major* iAC560 and *Trypanosoma cruzi* iSR215 model (Fig 5A and 5B) [15, 17]. Around 17% of the model reactions (exchange reactions excluded) are unique to the iAS142 model and have been newly curated. The comparisons indicate that the iAS142 model accounts for 36 novel intracellular reactions (17 compartmental + 19 intracellular transport) and 9 metabolites updated for their subcellular locations. These differences arise due to inappropriate assignment of reaction subcellular locations, unavailability of appropriate information for certain reactions, absence/existence of multiple subcellular location of enzymes, absence of important intracellular transport reactions reported through literature, and absence of mitochondrial inter-membrane space compartment in the iAC560 and iSR215 models, when compared to iAS142 model. The differences in intracellular reaction subcellular location between models are listed in Table 4. 16 distinct reactions are common to iAS142 and iAC560 and not to iSR215 and 2 distinct reactions are common to iAS142 and iSR215 and not to iAC560. 17 reactions are unique to the iAS142 model out of which 9 reactions have been newly added in the iAS142 model and 8 reactions have been updated for their reaction subcellular locations. These differences attribute the iAS142 model with a unique network structure that leads to prediction of biologically realistic scenarios, which have been discussed further in the article.

**Table 4:** Comparison of reaction subcellular locations within the iAS142 model with the iAC560 and iSR215 models.

Reaction (subcellular location)	Reaction abbreviation (iAS142)	iAS142	iAC560	iSR215	Confidence score in iAS142
Glucose-6-phosphate isomerase	PGIg1/PGIg2	Glycosome	Glycosome	Glycosome	5
	PGI1/PGI2^ǂ^	Cytoplasm		Cytoplasm	4
D-Lactate dehydrogenase	LDH_Dg^€^ ^,^ ^£^	Glycosome	Glycosome	Not considered	2
			Cytoplasm		
Citrate lyase	CSc*	Cytoplasm	Not considered	Not considered	5
Aconitase	ACONTm	Mitochondria	Mitochondria	Mitochondria	5
	ACONTc*	Cytoplasm			4
Isocitrate dehydrogenase	ICDHym	Mitochondria	Mitochondria	Mitochondria	5
	ICDHyc*	Cytoplasm			5
Fumarate hydratase	FUMm	Mitochondria	Mitochondria	Mitochondria	4
	FUMg	Glycosome	Glycosome	Glycosome	5
	FUMc^ǂ^	Cytoplasm		Cytoplasm	5
Glutamine synthetase	GLNSm^¥^	Mitochondria	Cytoplasm	Not considered	2
Asparaginase	ASNNg	Glycosome	Glycosome	Not considered	2
	ASNN*	Cytoplasm			1
glycine/serine hydroxymethyltransferase	GHMT	Cytoplasm	Cytoplasm	Not considered	5
	GHMTm*	Mitochondria			5
pyrroline-5-carboxylate reductase	P5CRr^€^ ^,^ ^£^	Cytoplasm	Mitochondria	Mitochondria	2
			Cytoplasm		
Glycine cleavage complex	GCCb^€^	Cytoplasm	Cytoplasm	Not considered	5
	GCCm*	Mitochondria			4
Ribokinase	RIBK*	Cytoplasm	Not considered	Glycosome	2
UDP sugar phosphorylase	USPx*	Glycosome	Not considered	Not considered	2
Aldose 1 epimerase	A1E^€^ ^,^ ^£^	Cytoplasm	Glycosome	Glycosome	2
			Cytoplasm		
Alanine aminotransferase	ALATA_Lm^€^ ^,^ ^£^	Mitochondria	Cytoplasm	Cytoplasm	2
			Mitochondria	Mitochondria	
Hexokinase	HEXg, HEX4g, HEX7g^€^	Glycosome	Glycosome	Cytoplasm	5
Fumarate reductase	FRDg^€^	Glycosome	Glycosome	Glycosome	5
	FRDm^€^	Mitochondria	Mitochondria		5
Ribose phosphate isomerase	RPI^€^	Cytoplasm	Cytoplasm	Glycosome	2
				Cytoplasm	
Ribulokinase	RBK_Dg^€^	Glycosome	Glycosome	Absent	2
pyrroline-5-carboxylate synthetase	G5SADs^€^	Cytoplasm	Cytoplasm	Mitochondria	—
glutamate-5-semialdehyde dehydrogenase	G5SDr^€^ ^,^ ^£^	Cytoplasm	Cytoplasm	Mitochondria	2
			Mitochondria		
Malic enzyme	ME1m^¥^	Mitochondria	Cytoplasm	Cytoplasm	2
				Mitochondria	
Acetyl-coA synthetase	ACS^¥^	Cytoplasm	Mitochondria	Not considered	5
Alcohol dehydrogenase	ALCDHm^¥^	Mitochondria	Cytoplasm	Not considered	2
NADPH cytochrome oxidoreductase	NADPH_CYOR*	Mitochondria	Not considered	Not considered	4
NADH:ubiquinone oxidoreductase	NADHDHmm^¥^ ^,^ ^#^	Mitochondrial membrane	Mitochondria	Mitochondria	5
				Cytoplasm	
ubiquinol-6 cytochrome c reductase	CYOR_u6mm^¥^ ^,^ ^#^	Mitochondrial membrane	Mitochondria	Mitochondria	5
cytochrome c oxidase	CYOO6m^¥^ ^,^ ^#^	Mitochondrial membrane	Mitochondria	Mitochondria	5
V-type H+-transporting ATPase subunit A	ATPSmm^¥^ ^,^ ^#^	Mitochondrial membrane	Mitochondria	Mitochondria	5
	ATPS	Cytoplasm	Acidocalcisome	Cytoplasm	5
			Cytoplasm		
phosphoglucomutase	PGMT^€^	Cytoplasm	Cytoplasm	Not considered	5
Aldehyde dehydrogenase	ALDD2Xm^€^	Mitochondria	Mitochondria	Not considered	5
6-phosphofructo-2-kinase	PFK26^€^	Cytosol	Cytosol	Not considered	5
Fructose-2,6-bisphosphate 2-phosphatase	FBP26^€^	Cytosol	Cytosol	Not considered	5

^**ǂ**^ **:** Reactions common to iAS142 and iSR215 but not iAC560

^€^: Reactions common to iAS142 and iAC560 but not iSR215

^£^: Reactions having single subcellular locations in iAS142 when compared to other models

^¥^: Reactions that have been updated for subcellular locations in iAS142

*: Reactions that have been newly added to iAS142

^#^: Reactions considered in a separate mitochondrial membrane compartment (b) uniquely within iAS142 model.

The mitochondrial membrane is not considered as a separate compartment in iSR215 and iAC560 models.

Further, to establish the uniqueness of the iAS142 network in predicting biologically realistic scenarios, we simulated the iAS142 model and the energy metabolism subset of the iAC560 model and compared the metabolic routes selected for glucose catabolism in both the models. As it can be observed from Fig 5C, the flux profiles of iAS142 and energy metabolism part of iAC560 models are highly similar (Spearman correlation coefficient (r) = 0.6672). Interestingly, flux through biomass reaction in iAC560 model (flux = 45.2) was relatively higher than flux through biomass reaction in iAS142 model (flux = 39.5). Further, the flux across individual reactions were considerably altered which we could capture from the scatter plot. The *L*. *major* model displayed a high rate of exchange of lactate from cytoplasm to extracellular and higher intake of pyruvate from extracellular to cytoplasm; whereas in the *L*. *infantum* model, there is neither any secretion nor uptake of pyruvate or lactate (Fig 5D).

As there are significant differences between the gene-protein-reaction associations between the two models (Table 4), the flux distribution also varied between the two models. One of the major differences observed was the increased dependence of the iAC560 model on lactate fermentation and hence pyruvate uptake, instead of succinate fermentation which was observed in the iAS142 model. This major difference was due to presence of cytoplasmic lactate dehydrogenase and a cytoplasmic alanine aminotransferase in the *L*. *major* iAC560 model, which was not curated for the *L*. *infantum* iAS142 model. Performing FBA on the *L*. *major* iAC560 energy metabolism subset, it could be observed that cytosolic lactate dehydrogenase and alanine transaminase form a cycle along with their glycosomal (lactate dehydrogenase) and mitochondrial (alanine aminotransferase) counterparts respectively. This produces a sink for the pyruvate that is produced from mitochondrial alanine aminotransferase, pyruvate dehydrogenase reactions and extracellular pyruvate uptake. This cycle causes fermentation of lactate to be preferred over succinate. This requirement of lactate production leads to a corresponding higher activity of the pentose phosphate pathway (PPP) reactions to maintain redox balance within the glycosome in the iAC560 model. This leads to an over production of CO_2_ from the 6-phopshogluconate dehydrogenase reaction which is released outside the cell to the extracellular (Fig 5D). On the contrary, in the *L*. *infantum* iAS142 model, where the cytoplasmic lactate dehydrogenase and the cytoplasmic alanine aminotransferase reactions are absent, succinate fermentation is preferred to maintain redox balance within the glycosome, and thus, drives CO_2_ uptake rather than release. Hence, the iAS142 model shows a minor uptake of CO_2_ from extracellular to cytoplasm (Fig 5D). The comparison between the flux profiles of the reactions from the two models leading to production of lactate revealed a significant difference (p<0.01). The reactions considered for this comparison were pyruvate extracellular uptake, pyruvate glycosomal transport, mitochondrial alanine transaminase, glycosomal lactate dehydrogenase, glycosomal lactate transport, extracellular lactate transport and the pentose phosphate pathway reactions. Further, succinate fermentation was recently shown to be a bare essential for the *Leishmania* parasite to mediate glycosomal redox balance and hence, energy metabolism [18], further supporting the accurate curation and annotation of the iAS142 model.

### Effect of amino acids supplemented with glucose on model reaction fluxes

**Fig 6:**
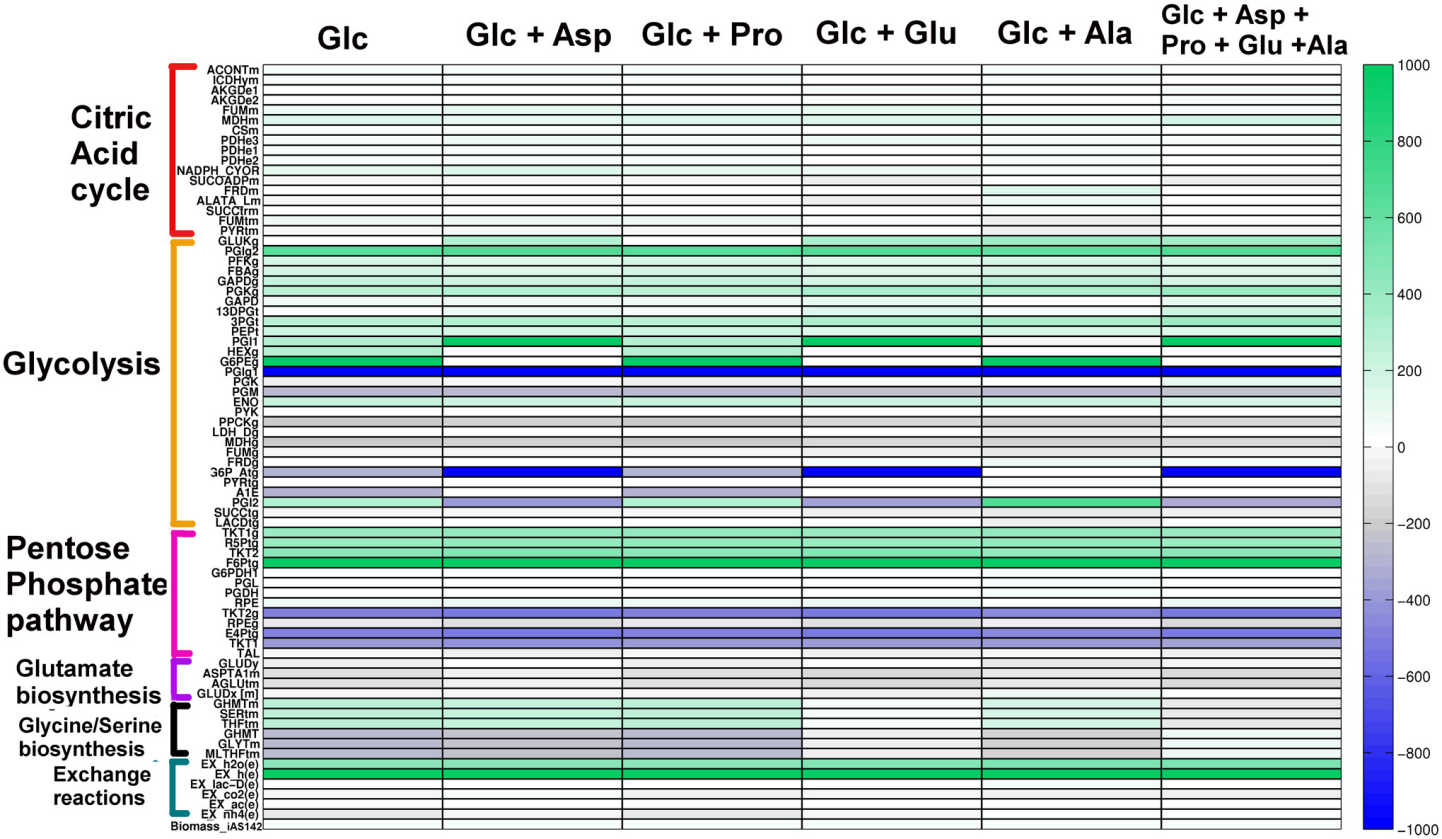
Effect of amino acids when coupled with glucose uptake: Heatmap to visualize differences in flux through crucial reactions for different scenarios. The amino acids were allowed to enter one at a time along with glucose to identify different metabolic routes operational in diverse conditions. Glc = Glucose, Asp = Aspartate, Glu = Glutamate, Ala = Alanine, Pro = Proline.

*L*. *mexicana* developmental stages were shown to co-utilize glucose and a few non-essential amino acids like glutamate, aspartate, alanine and proline, when cultured in a completely defined medium consisting of a range of carbon sources [18, 19]. In order to understand the importance of these non-essential amino acids in energy metabolism of *L*. *infantum*, each one of them was supplemented with glucose uptake, together as well as individually. Performing FBA for each of these cases led to the generation of different flux distributions, displayed in the form of a heat map (Fig 6). The default simulation results where glucose is the only nutrient are also shown for comparison.

It was observed that, when different non-essential amino acids supplement glucose as nutrients, fluxes of glycolysis, glutamate biosynthesis and glycine/serine biosynthesis reactions vary the most. Clearly, the fluxes through pentose-phosphate pathway and the citric acid cycle reactions are fairly constant, suggesting their housekeeping functions within the cell. Most importantly, the reaction fluxes of TCA cycle reactions lie in the small range of -200 to 200, suggesting that molecular feed into the TCA cycle is quite low. At the same time, majority of glycolytic reactions retain higher flux values, between ranges 800 to 1000 and -800 to -1000 for all the scenarios. This is a very important aspect of *Leishmania* metabolism, which suggest that utilization of glucose is always very prominent albeit the presence of other carbon sources. Also, growth was reduced to zero when the exchange of glucose was constrained to zero i.e. glucose entry was restricted, in spite of the abundance of amino acids. This strongly suggested that glucose is the prime essential substrate for parasite growth. Here it is very important to keep in mind that the iAS142 model does not take the fatty acid metabolism into consideration. Therefore, an acetyl CoA uptake was added to the model as it is the refined product of fatty acid catabolism. But even after this, there was no growth in the absence of glucose, arguing that cell could not metabolize acetyl CoA in place of glucose.

The biomass flux showed a 1–1.5 fold increase with addition of amino acids’ uptake along with glucose uptake (see S4 Table). In the optimal solution, the flux through amino acids uptake when supplemented with glucose uptake, was in the order—proline uptake < aspartate uptake < glutamate uptake < alanine uptake (see S1 Text and Panel A in S1 Fig). Similarly for each of these situations, the flux through biomass production/growth rate was in the sequence—glutamate uptake > alanine uptake > asparte uptake > proline uptake (see Section E in S1 Text and Panel B in S1 Fig). This result suggested that glutamate and alanine are the most preferred substrates after glucose, as they lead to maximum increase in biomass in comparison to other amino acids. On the contrary, proline is slowly taken up by the cell from environment compared to other amino acids when supplemented with glucose. Moreover, when all amino acid fluxes were simultaneously allowed along with glucose for uptake, there was zero or no flux observed through aspartate and proline uptake in the optimal solution. This further strengthens the argument that glutamate and alanine are preferred by *L*. *infantum* over aspartate and proline, despite their equal abundance in the environment. In all the situations created, with respect to the biomass equation considered, it can be observed that glutamate biosynthesis reactions play a major role in driving ATP generation, by activating the TCA cycle via succinate fermentation. This phenomenon was also observed through isotopomer profiling experiments [18], qualitatively validating the strength of iAS142 in predicting the metabolic routes preferred by the parasite to catabolize glucose and its coupled non-essential amino acids.

### Choice of biomass objective function affects model flux distribution

**Fig 7:**
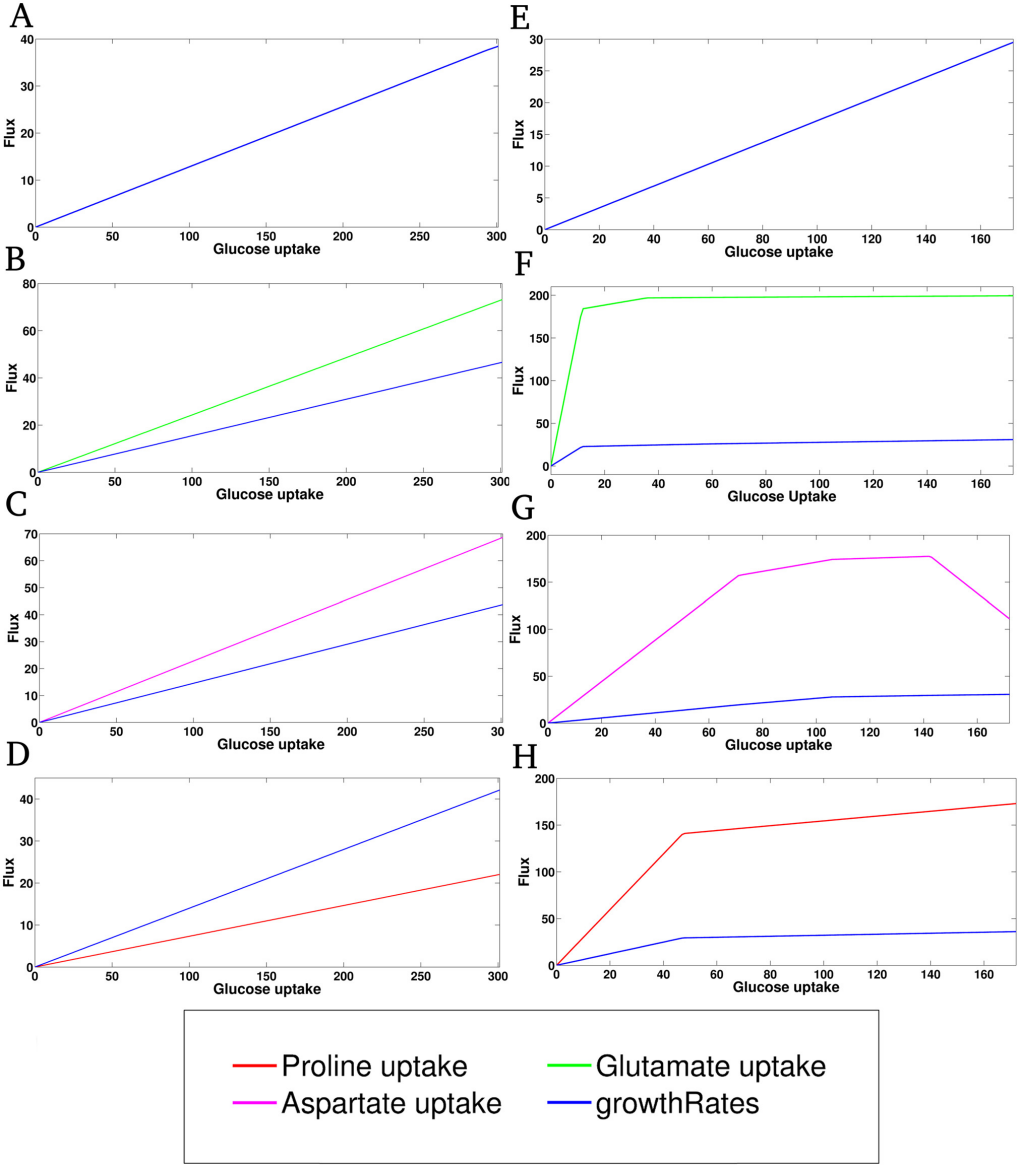
Comparison of the iAS142 biomass reaction with the iSR215 biomass reaction. A) and E) represent situations where glucose is the sole substrate with respect to the two biomasses respectively. In all the other cases an amino acid supplements glucose uptake, which can be identified by their respective colors. B), C), and D) depict the effect of variation in glucose uptake on fluxes through glutamate, aspartate and proline uptake respectively, using the iAS142 biomass reaction. Effect on biomass growth rate is also recorded in each case. F), G), and H) demonstrate the same, but when iSR215 biomass reaction is used.

In a previous study that modeled the energy metabolism in *Trypanosoma cruzi* (iSR215), an objective function (Tryp_biomass) derived from the biochemical data of *Bacillus subtilis* was used for performing flux balance analysis on the model [15]. Whereas, the biomass objective function used for performing FBA in this study on the iAS142 model (Biomass_iAS142) was derived from the aforementioned 13-C isotope enrichment data. Therefore, both these biomass objective functions were taken separately, one at a time and optimized while performing FBA on the iAS142 model (see S5 Table) and the results were compared. In the previous section, it was observed that glucose uptake is essential for uptake of non-essential amino acids. Thus, a robustness analysis of the amino acid uptake and biomass itself with respect to varying glucose uptake was performed to visualize any deviation resulting from the usage of different biomass objective functions (Fig 7). The upper and lower bounds of glucose uptake in the model for each biomass reaction was fixed to a value that was incrementally increased within the bounds of 0 to the optimal value of glucose uptake (optimal flux value = 310) obtained through the default FBA simulation. As it is very much evident from the Fig, the biomass growth rate and amino acids uptake varies linearly with glucose uptake for all situations when Biomass_iAS142 is used (Fig 7A to 7D). On the other hand using the iSR215 biomass, linear relationship between growth rate and glucose uptake could be perceived only for the situation, where glucose was used as the sole carbon source (Fig 7E). For rest of the other cases where amino acids supplemented glucose, both amino acid uptakes and biomass were in a piece wise linear relationship with glucose uptake (Fig 7F to 7H).

### Stage specific energy metabolism of *Leishmania infantum*

As discussed before, the *Leishmania* parasite is able to persist in two extremely opposite environments, the sandfly gut and the human macrophage by switching between the promastigote and amastigote developmental stages, each stage adapted to survive in a particular environment. In order to identify the metabolic routes by which *L*. *infantum* achieves this feat, we recreated model scenarios that might represent the promastigote and amastigote metabolic states (see Section F in S1 Text). Details behind re-creation of these stage specific scenarios are discussed in the Methods section. The pathway specific distribution of reaction fluxes in both scenarios is available in S6 Table.

**Fig 8:**
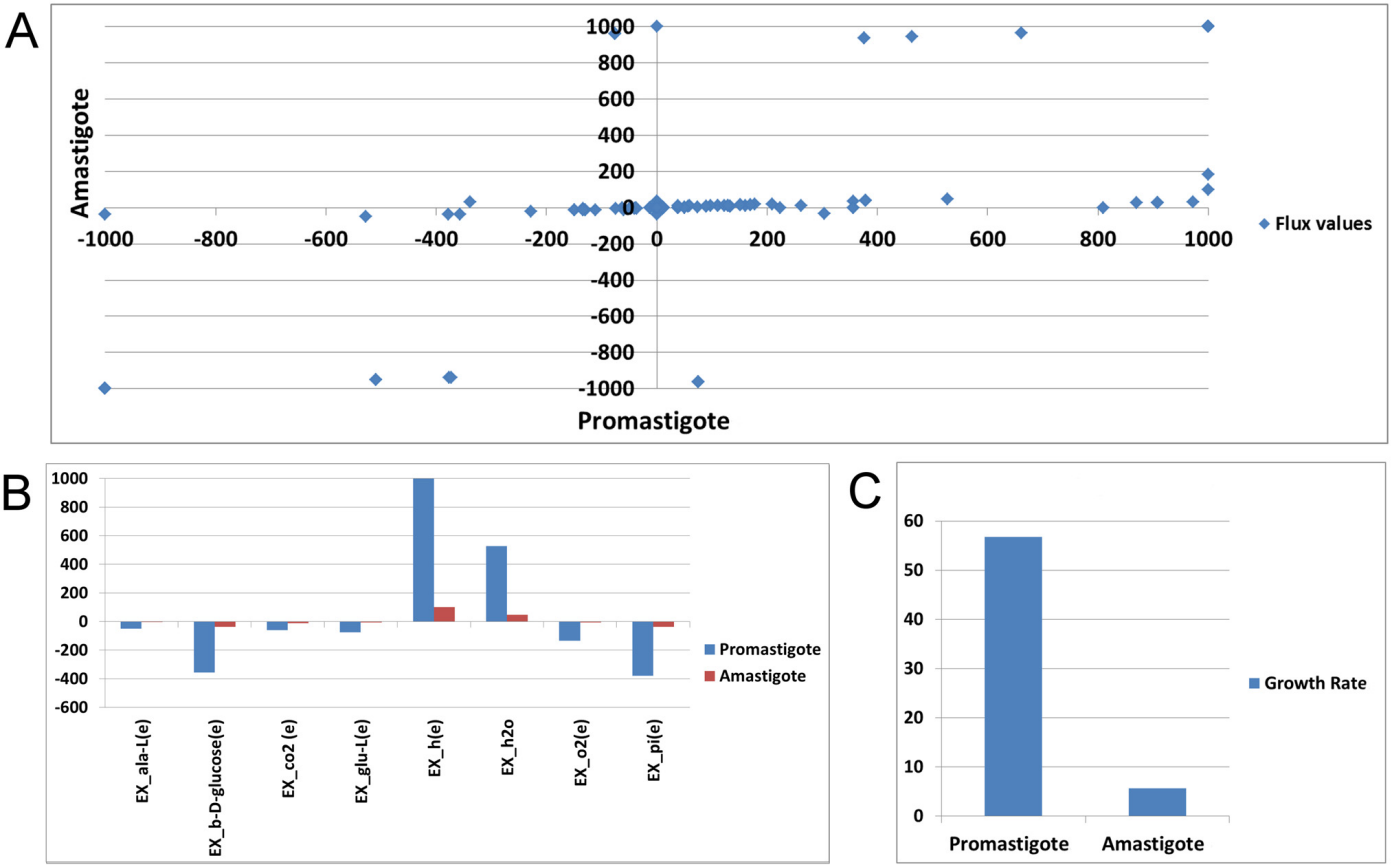
Comparison of flux distributions between the promastigote and amastigote scenarios. A) Scatter plot showing the variation of fluxes from the promastigote to amastigote forms B) Bar plot showing differences between overflow metabolism in the two developmental stages of *L*. *infantum* C) Bar plot showing the differences in promastigote and amastigote growth as predicted from the model.

Flux through around 90% of reactions in amastigote metabolism was substantially reduced when compared to promastigotes (Fig 8A). Around 113 reactions out of the total 237 reactions in the model completely shut down (no flux) in the amastigote flux profile (Fig 8A, S6 Table, S2 and S3 Figs). Around 11 reactions were uniquely functioning in amastigotes and absent in promastigotes (see S6 Table). In the amastigote metabolism, glucose uptake reduced by 10 fold and glutamate uptake rate reduced by almost 15 fold when compared to the promastigote profile justifying the glucose-deficient conditions in which the amastigote survives (Fig 8B). Also uptake of other non-essential amino acids, like aspartate and alanine, were considerably reduced. A similar reduction in glucose and non-essential amino acid uptake rate was previously reported in the *L*. *mexicana* amastigotes in comparison with the promastigote stage [5, 19]. The metabolic flux through the glycolysis, tricarboxylic acid cycle (TCA) and the pentose phosphate (PPP) pathways in the amastigote metabolic state significantly drops (see Section F in S1 Text and S4 Fig). This leads to a reduction in the secretion of overflow metabolites as well (Fig 8B). Since the real phenotype data specific to *L*. *infantum* was unavailable, the stage specific reaction fluxes observed from our model could be qualitatively compared to the behavior of enzymes of carbohydrate metabolism in the closely related visceral species *L*. *donovani* [73]. The change in the reaction fluxes of these pathways qualitatively relate to the changes observed in the specific activity of enzymes required for carbohydrate metabolism in the amastigote forms of the visceral *L*. *donovani* species [73]. Even though the information used for the above comparison was not *L*. *infantum* specific, as they are closely related species, probably there would not be a drastic difference in the energy metabolism between these species. These comparisons have been further explained in Section F of S1 Text.

Also, to our surprise, the optimal solution for oxygen exchange suggested a reduction in oxygen intake in the amastigote scenario as compared to the promastigote scenario, probably signifying the adaptation of amastigote metabolism to the hypoxic environment of the macrophage. As amastigotes reside in an acidic environment, covered by abundance of hydrogen ions, it is quite intuitive that in a highly acidic environment, the amount of hydrogen transferred from the cell to the environment would be very low. Hence, amastigote metabolism was adapted to a high reduction in hydrogen ion release to the environment (Fig 8B). The observed reductions in glucose and oxygen uptake perhaps led to rapid decrease in amastigote growth rate (Fig 8C).

### Simulation of chemotherapeutic intervention in amastigotes

Knockout studies in the iAS142 model revealed important reactions that were deemed to be absolutely essential for parasite growth (See “Reaction knockout analysis and predictions” sub section in the Result section). These predicted targets were further used to simulate the effect of a chemotherapeutic in inhibiting an enzyme in the amastigote stage (see Methods Section for details). The flux values of the target reactions were varied from its optimal flux evenly up to zero, performing a separate FBA simulation for every flux value change; capturing the corresponding growth and ATP synthesis rates. While performing this, flux of only one target reaction was varied at a time.

**Fig 9:**
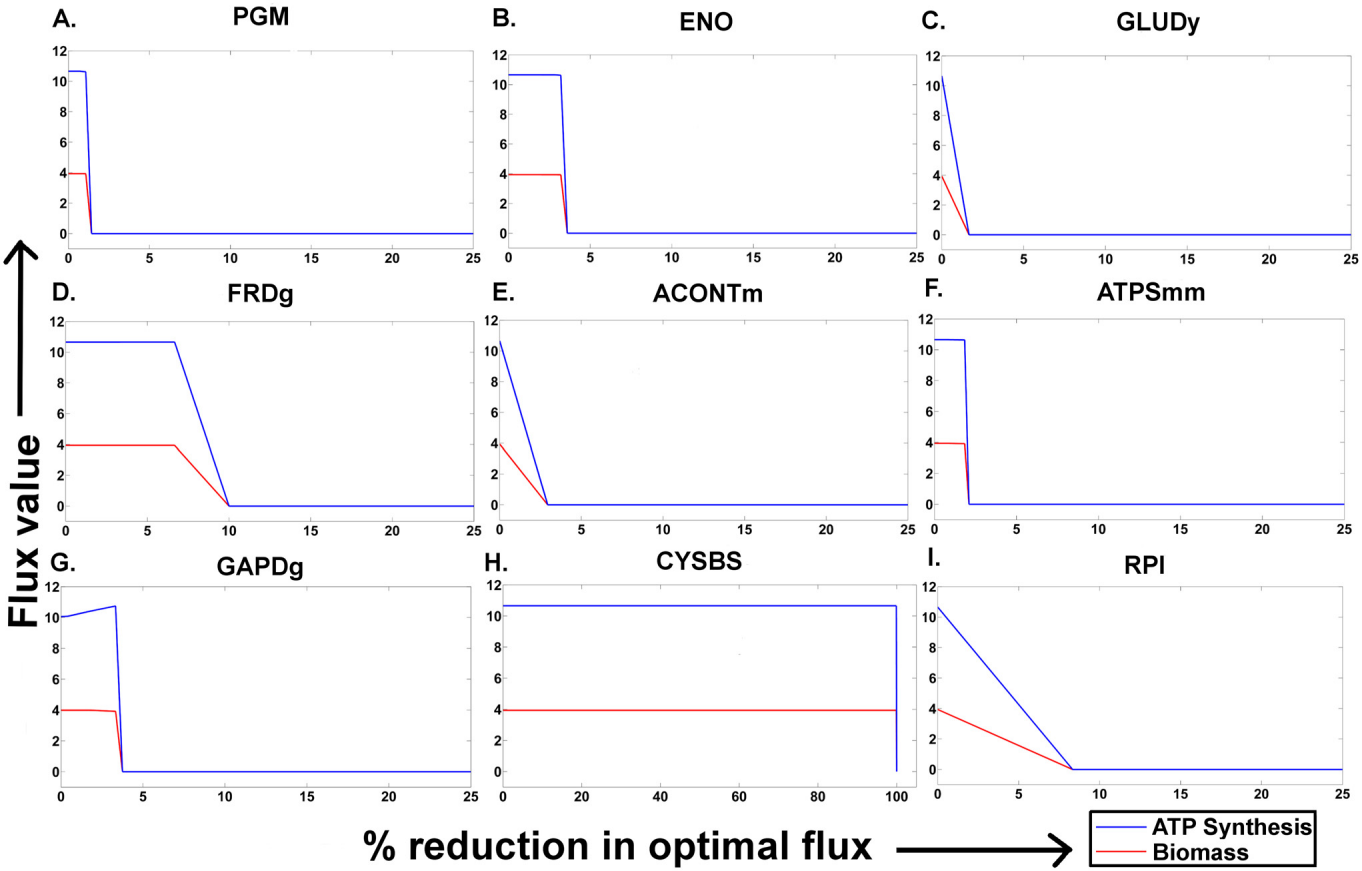
Chemotherapeutic intervention scenarios and targeting essential reactions. Percentage reduction in the optimal flux magnitude of A) Phosphoglucomutase (PGM) B) Enolase (ENO) C) cytoplasmic glutatmate dehydrogenase (GLUDy) D) glycosomal furamate reductase (FRDg) E) mitochondrial aconitase (ACONTm) F) mitochondrial membrane ATP synthase (ATPSmm) G) glycosomal glyceraldehyde-3-phosphate dehydrogenase (GAPDg) H) Cystathione beta lyase (CYSBS) I) Ribose phosphate isomerase (RPI) and its effect on growth (red curve) and ATP synthesis (blue curve).

Assuming that reaction flux relates to the biochemical activity of an enzyme in catalyzing a reaction, a percentage reduction in the optimal flux value of a particular reaction may simulate percentage partial/complete inhibition of that enzyme, if an inhibitor is used. Fig 9 displays the effect of percentage reduction of flux through previously identified essential reactions on growth and ATP synthesis. Although each of these targets has been qualitatively identified through model reaction knockouts, a number of quantitative differences in growth rate and ATP synthesis could be evaluated between the targets when its activity/flux was partially reduced. A reduction of 2% and above in the optimal fluxes/actvities of phosphoglucomutase (PGM) and glutamate dehydrogenase (GLUDy) reactions lead to immediate decline in growth and ATP synthesis (Fig 9A and 9C). On the contrary, for cystathione beta lyase (CYSBS), a 100% inhibition of CYSBS activity leads to zero growth (Fig 9H). Also, through this analysis, inhibition thresholds for any target-specific chemotherapeutic to obtain zero growth could be predicted. For instance, considering cytoplasmic glutamate dehydrogenase (GLUDy) as a probable target, from Fig 9C one can point out that around 2% inhibition in the activity/flux of the glutamate dehydrogenase reaction could lead to permanent depletion of parasite growth and ATP synthesis. Similarly, a 9% reduction in the activity/flux of the ribose phosphate isomerase (RPI) reaction would lead to absolute depletion in parasite growth and ATP synthesis (Fig 9I). A similar rationale can be used to describe flux reduction in other reactions. Similar to the mentioned reactions, a reduction of around 4% in enolase (ENO), 10% in glycosomal fumarate reductase (FRDg), 4% in mitochondrial aconitase (ACONTm), 3% in mitochondrial membrane ATP synthase (ATPSmm), 4% in glycosomal glyceraldehyde 3 phosphate dehydrogenase (GAPDg) leads to zero growth and ATP synthesis in the amastigote (Fig 9B, Fig 9D to 9H respectively).

## Discussion

In this paper, we present the first constraint-based model reconstruction and analysis of energy metabolism in *Leishmania infantum*, the causal organism of a vital neglected tropical disease, visceral leishmaniasis. From our simple model-based study, we explore the different facets of the *Leishmania infantum* energy metabolism and reveal novel features of parasite metabolism under varying environmental conditions. The model accounts for central metabolic processes that include ATP synthesis and production of key intermediates that are essential for biomass production/growth. Furthermore, the model consists of reactions characterized into 5 different compartments—four cellular—the glycosome (characteristic compartment of the Trypanosomatids), the mitochondrion, the mitochondrial intermembrane space and the cytosol and an extracellular for exchange and transport of metabolites from the cell to the environment. The main point to note here is that none of the previous constraint-based models have considered the mitochondrial intermembrane space compartment [15, 17, 51]. The inclusion of this compartment was imperative, since the proton gradient established by oxidative phosphorylation is coupled to ATP synthesis between the mitochondrion and the intermembrane space, and not with the cytosol. Based on relevant proof from literature and appropriate sequence analysis, reactions have been assigned to specific subcellular locations in the model (giving a high confidence to those reactions having strong literature proof). As a result, around 54 reactions could be assigned to their appropriate locations with high confidence.

The iAS142 metabolic network was analyzed using flux balance analysis with respect to a novel metabolic demand/biomass reaction; developed using 13C isotopic enrichment data acquired from *L*.*mexicana* promastigotes grown in medium containing 13C labeled glucose [19]. None of the previous FBA constraint-based models [15–17] have used 13C isotope data for developing the biomass reaction. From the model analysis and validations, the strength of the iAS142 biomass in accurately predicting the intricate details of *L*. *infantum* metabolism could be fairly demonstrated. Further, the iAS142 biomass was compared with the biomass objective obtained from a previously published *Trypanosoma cruzi* iSR215 model to enlighten the influence of biomass objective on reaction flux distribution [15]. The objective behind this analysis was to highlight the importance of the biomass reaction and the variation in reaction fluxes on considering a different biomass function. This also brings forward the amount of scrutiny which should be maintained while performing modeling studies using FBA.

A two-tier validation of the model was performed to assess its importance in predicting the actual metabolic aspects as perceived from various experimental studies. The first stage of model validation was performed through prediction of growth phenotypes using *in silico* reaction knockout analysis and comparison with experimentally known phenotypes. With respect to available knockout phenotype information in *Leishmania*, the model could predict the corresponding known growth phenotypes associated with 7 reaction knockouts with an absolute accuracy of 100%. Apart from validation purposes, the reaction knockout study gave a distinct biological insight into the crucial role played by every reaction in *L*. *infantum* energy metabolism. Even though it is inappropriate to compare the model predictions with *Trypanosoma* species that show stage specific differences in metabolism and reside in different environments than *Leishmania*, the model predictions were compared with available non-stage specific *Trypanosoma* knockout phenotype data only to further stress on the fact that the model was indeed *Leishmania* specific giving biological reasoning behind every single gene deletion phenotype predicted from the model. A total of 61 lethal reactions were identified through single reaction deletions in the network and 55 non trivial lethal reaction pairs were proposed to be essential through double reaction deletions. Each one of these deletions constitutes a promising drug target and an experimentally testable hypothesis, which we considered further to show the chemotherapeutic intervention scenarios for 9 reactions predicted to be essential for parasite growth from the model reaction knockout analysis.

Even though the model predictions are usually validated through reaction knockouts, there are three important shortcomings in validating a constraint-based model through reaction knockout studies [15]. First, due to unavailability of full knockout data specific to *L*. *infantum*, many knockout validations were not made from *L*. *infantum* data alone, but also considering data obtained from other Trypanosomatid species. Second, as the model considered here is not a genome-scale model, there might be other essential pathways that might be connected to core energy metabolism but not considered in the model. Hence, it is possible that the model might predict a reaction to be non-lethal, even though in the whole genome scale context it might be lethal or vice versa. Third, the experimental knockout data for validation purposes were collected from heterogeneous studies that might have considered different experimental conditions or media to generate knockouts, which are not explicitly implemented in our model. Hence, we prefer to use the model reaction knockout analysis as a predictor of essential genes required for parasite growth rather than a basis for validating our model.

In order to overcome the limitations of validation through reaction knockout studies, by performing a robustness analysis with respect to changes in oxygen uptake in our model, we attempted to validate the model by identifying model conditions to discern the secretion of overflow metabolites. *Leishmania* is known to exhibit an overflow metabolism during which it secretes substantial amount of succinate, acetate, pyruvate, CO_2_ and small amounts of lactate [57, 58]. This secretion is largely controlled by differences in the oxygen and glucose concentrations in the medium. With variation in both glucose and oxygen uptake, we were able to observe secretion of all the aforementioned overflow metabolites. More specifically, model robustness analysis discovered the secretion of lactate via the D-lactate dehydrogenase reaction which was one of the most important aspects that verify the model indeed to be *Leishmania* specific. Sequence analysis and experiments indicate absence of a functional D-lactate dehydrogenase in *Trypanosoma* species [6, 72, 74]. Although, *Trypanosoma* species have been shown to demonstrate L-lactate secretion instead of D-lactate, the pathway for this mechanism is still unknown [6]. Thus, with respect to production and secretion of *Leishmania* specific overflow metabolites, the model could be appropriately validated.

Promastigote and amastigote specific metabolic scenarios were created from the model so as to observe the stage specific differences in the energy metabolism of *L*. *infantum*. 13C isotope resolved metabolomics in *L*. *mexicana* developmental stages revealed a reduction of glucose uptake, glutamate uptake and secretion of overflow metabolites in axenic amastigotes as compared to promastigotes [19]. Similarly, after incorporating the amastigote scenario in our model, we could capture the reduced rate of glucose and glutamate uptake and reduced exchange of overflow metabolites with the environment experimentally observed in amastigotes. With respect to other carbon sources, uptake of other non-essential amino acids like aspartate, proline and alanine also reduced. As a result, a large reduction in glycolysis, TCA cycle, ATP synthesis, and amino acid metabolism reaction fluxes could be observed in the amastigote scenario. Also, a distinctive reduction in extracellular oxygen intake and hydrogen ion release was observed probably signifying the parasite’s adaption to the hypoxic and acidic environment of the human macrophage. Despite the considerable reduction in reaction fluxes, flux through transketolase reactions in the amastigote scenario was high when compared to the promastigote scenario suggesting the hyperactivation of pentose phosphate shunt in fulfilling the demand for glucose-6-phosphate under glucose-deficient conditions. Also, in both the promastigote and amastigote metabolic scenarios, closure of glucose uptake even in abundance of other amino acids led to abolishment of parasite growth signifying that glucose is the most important and essential carbon source preferred by both the developmental stages of the parasite though they exist in completely different environments.

The reaction knockout studies could predict the enzymes of succinate fermentation as essential for the organism’s growth suggesting the role of succinate fermentation pathway in maintaining a redox balance within the glycosome by regenerating the NAD molecules consumed by the enzymes of upper glycolytic pathway [6, 18]. Furthermore, for model simulations both with only glucose and glucose supplemented with amino acids, the results exhibited the replenishment of the TCA cycle through C4 dicarboxylic acid intermediates like malate, fumarate and succinate produced via succinate fermentation pathway, proving the importance of glycosomal succinate fermentation in TCA anaplerosis. By tracing the flux distribution through the model reactions, we could hypothesize that the major reason for activation of succinate fermentation was the demand for synthesis of glutamate through the glutamate dehydrogenase and aspartate aminotransferase reactions. The same was observed through 13C resolved energy metabolism in *L*. *mexicana* promastigotes [18]. It is important to note here that it is the network structure of the *L*. *infantum* energy metabolism as implemented in the iAS142 model that governs this metabolic behavior. Also, it is through the same metabolic route that the cellular energy maintenance of the parasite is met. This particular route was also, preferred in both the promastigote and amastigote scenarios of energy metabolism that were recreated in the model. Fascinatingly, model reaction knockout studies also predicted cytosolic NADPH–dependent glutamate dehydrogenase to be crucial for *Leishmania infantum*. All this information point towards the role played by succinate fermentation and glutamate biosynthesis in driving energy metabolism further establishing the fact, that the enzymes of succinate fermentation and glutamate biosynthesis could possibly be novel therapeutic targets for the *L*. *infantum* parasite.

As a part of rational drug design in *Leishmania* species, identification of novel drug targets in an early discovery phase has become increasingly important so as to design new small molecule inhibitors that can serve as potential drug candidates against the parasite [22, 24]. The amastigote developmental stage is the most sought after for drug discovery as it is the stage that is the cause of infection in humans. Gene essentiality studies in metabolic networks identify probable chemotherapeutic targets, by qualitatively assessing the role of a particular protein played in growth of the organism [17, 75–77]. To further demonstrate the applicability of FBA-based model analysis in quantitatively predicting and categorizing essential reactions to be good drug targets, we incorporated *in silico* chemotherapeutic intervention scenarios within our model-presumed amastigote stage for 9 essential reactions predicted by *in silico* reaction knockout analysis carried out in the iAS142 model. By partially reducing the optimal flux value of each of these individual reactions in the amastigote scenario, a minimum inhibition threshold a chemotherapeutic needs to exert on each reaction to achieve zero parasite growth was identified through this simulation. Accordingly, the predictions indicated that for glutamate dehydrogenase and phosphoglucomutase reactions in comparison to the other 7 reactions, a minimal reduction in enzyme activity/reaction flux brought about an immediate decline in the parasite growth suggesting their choice as an important drug target.

## Conclusion

In this study, we presented a reconstructed metabolic network for *Leishmania infantum*. As compared to other metabolic reconstruction studies in Trypanosomatids, our model accounts for a unique compartment like the mitochondrial intermembrane space and 36 novel intracellular reactions assigned to appropriate subcellular locations. In the process of model reconstruction, annotation of certain genes was refined and corresponding reactions to their appropriate subcellular locations were dispensed. The model was analyzed using a biomass objective function based on 13C isotope enrichment data; this also, being unique to the iAS142 model. With the aid of reaction knockouts, robustness analysis of overflow metabolites and observations in the effects of different amino acids on parasite energy metabolism, innumerable features of parasite metabolism could be extensively explored.

Analyses of the model revealed important roles of succinate fermentation, an active TCA cycle, and glutamate biosynthesis on parasite survival and growth. Also, single and double reaction deletions in the model led to identification of promising drug targets that can be experimentally validated in the future. The promastigote and amastigote specific metabolic states created from the model suggest that amastigotes display a glucose-sparing metabolism by reducing the uptake of glucose from the environment. The *in silico* therapeutic intervention scenarios created in the model specific for the amastigote could reveal an application of a FBA-based model in categorizing drug targets predicted to be essential from reaction knockout analysis for a parasite. Thus, from our simple but conceptually intensive model-based study, we could explore the different facets of the *Leishmania infantum* energy metabolism and substantiate the importance of such integrative metabolic reconstruction studies in unearthing the diverse features of parasite metabolism.

## Supporting Information

S1 Fig Effect of amino acids’ uptake supplemented with glucose. The bar plots show the differences in flux profiles of amino acids supplemented with glucose. A) Bar plot showing absolute flux values of amino acid uptakes when supplemented with glucose for which we get optimum biomass B) Bar plot showing growth rates when glucose uptake is supplemented with corresponding amino acids.
(TIF)

S2 Fig Promastigote metabolism. Flux distributions are overlaid on pathway map. The map distinctly shows that glycolysis, pentose phosphate pathway, TCA cycle and glutamate biosysnthesis reactions are constitutively activated in promastigote metabolism with high flux through every reaction. Also, there is an increased rate of ATP synthesis, glucose uptake and uptake of non-essential amino acids.
(TIF)

S3 Fig Amastigote metabolism. Flux distribution overlaid on pathway map. The map distinctly shows that glycolysis, pentose phosphate pathways and glutamate biosysnthesis reactions are constitutively activated in amastigote metabolism but, the reaction fluxes are considerably reduced as compared to promastigote metabolism. Also, there is reduced rate of ATP synthesis, glucose uptake and uptake of non-essential amino acids.
(TIF)

S4 Fig Stage specific energy metabolism. Bar plots showing differences between the amastigote and promastigote specific metabolism at the pathway level. A) differences observed in the glycolytic reactions in the amastigote and promastigote stages B) differences observed in the TCA cycle reactions in the two stages C) differences in the pentose phosphate pathway observed between the two stages D) differences between other important reactions in both the stages
(TIF)

S1 File The iAS142 reconstruction. This is a zip file that contains the iAS142 *L*. *infantum* energy metabolism reconstruction saved in both SBML and.mat file formats.
(ZIP)

S1 Table Data curated for model reconstruction. This excel file contains the information curated for the iAS142 model reconstruction. It contains information about the reactions considered in the model along with the information about the genes and proteins associated with the reactions and their subcellular locations. A column for the confidence score for every reaction has also been included. The PubMed ID of the references is provided beside every reaction entry. The list of references curated are listed at the end of the excel file.
(XLS)

S2 Table Confirmation of subcellular location by sequence based prediction of cellular signals in homologues. This excel file contains the set of enzymes whose subcellular localizations were confirmed by detection of cellular signals in their homologous protein sequences obtained as a result of performing BLAST against the RefSeq database excluding sequences from Genus *Leishmania*.
(XLS)

S3 Table *In silico* reaction knockout predictions from the iAS142 model. This excel file contains the detailed information of the growth phenotypes predicted from the model by keeping bounds of glucose and oxygen fixed to their optimal values. The predictions have been compared with the experimentally observed growth phenotypes listing their references beside growth phenotype prediction of every reaction considered in the model.
(XLS)

S4 Table Effect of amino acids’ uptake supplemented with glucose uptake. The flux profiles of every non-essential amino acid (aspartate, proline, glutamate, alanine) supplemented with glucose separately and in combination are given in separate columns in the excel file. In order to give a comparison, the flux profile generated for the only glucose situation as a control has also been given.
(XLS)

S5 Table Comparison of flux profiles generated from the model for Biomass_iAS142 and iSR215 biomass. The excel file contains the flux distributions obtained from the model by considering the two biomass objective functions. This was done to explain the importance of choosing a biomass objective and how it might affect the model predictions.
(XLS)

S6 Table Comparison of promastigote and amastigote metabolism. Flux profiles generated in the promastigote and amastigote specific scenarios have been given in the excel file. The model was constrained for the uptake of glucose and non-essential amino acids in both the scenarios and flux profiles were generated using FBA.
(XLS)

S1 Text This text contains 5 sections namely A, B, C, D, E and F. Section A discusses about the formulation of the biomass objective function from 13C isotopic enrichment data. The other sections relatively support the results of the model described in the main article. It also contains three tables, Table SB, Table SC and Table SD, which are relevant to support the model analyses discussed in the methods and results section of main article.
(DOC)
