# Supplementary figures and images for: Dissecting Leishmania infantum Energy Metabolism - A Systems Perspective
Source: PLoS One. 2015 Sep 14;10(9):e0137976. doi: 10.1371/journal.pone.0137976 (PMC4569355; doi:10.1371/journal.pone.0137976)

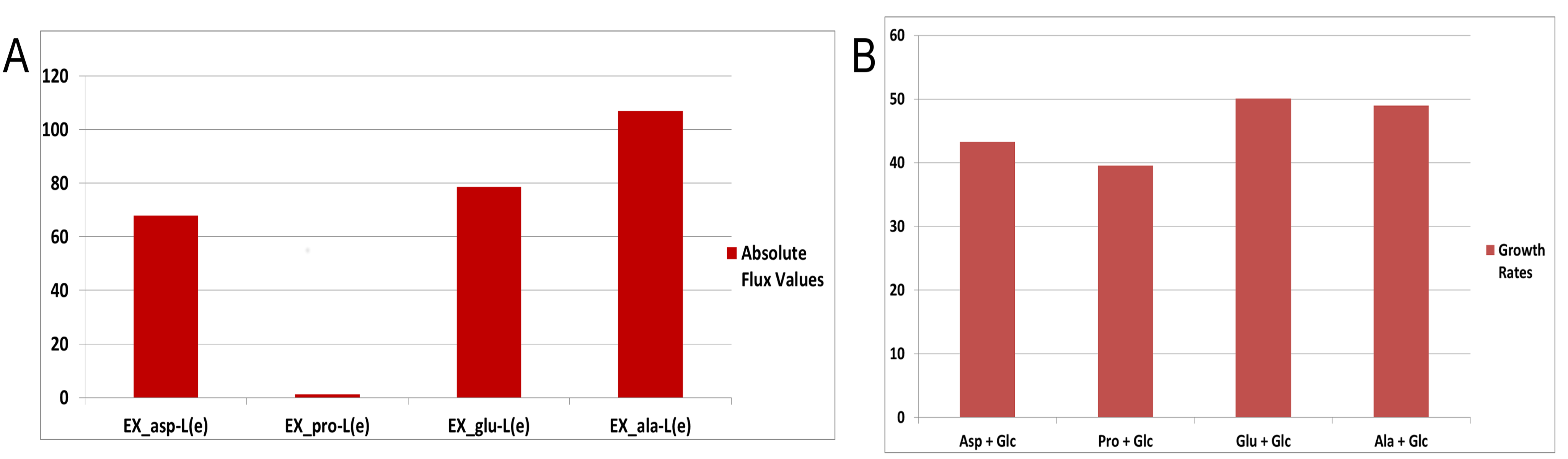

Supplement: S1 Fig — The bar plots show the differences in flux profiles of amino acids supplemented with glucose. A) Bar plot showing absolute flux values of amino acid uptakes when supplemented with glucose for which we get optimum biomass B) Bar plot showing growth rates when glucose uptake is supplemented with corresponding amino acids. (TIF) [file pone.0137976.s001.tif]

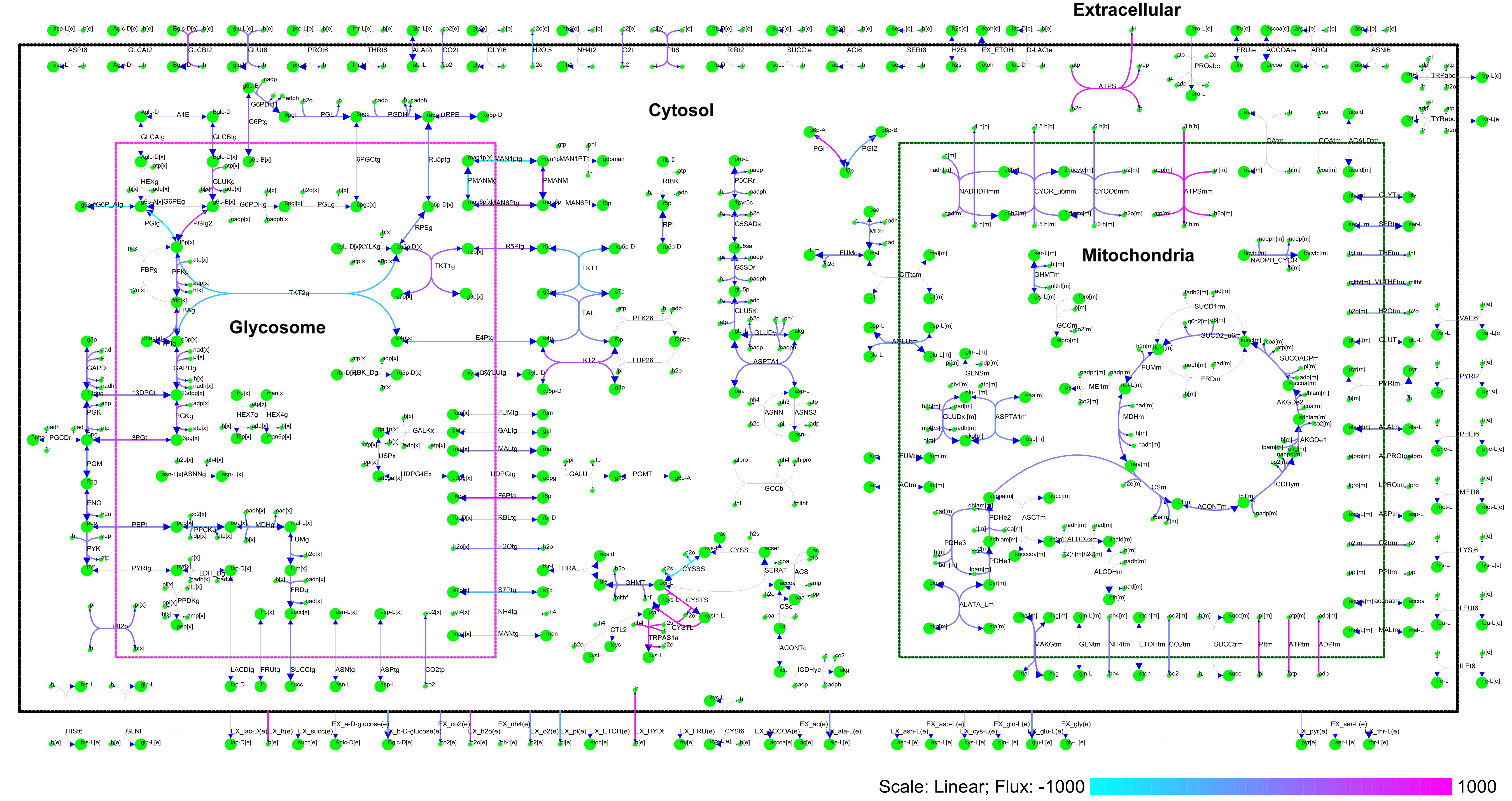

Supplement: S2 Fig — Flux distributions are overlaid on pathway map. The map distinctly shows that glycolysis, pentose phosphate pathway, TCA cycle and glutamate biosysnthesis reactions are constitutively activated in promastigote metabolism with high flux through every reaction. Also, there is an increased rate of ATP synthesis, glucose uptake and uptake of non-essential amino acids. (TIF) [file pone.0137976.s002.tif]

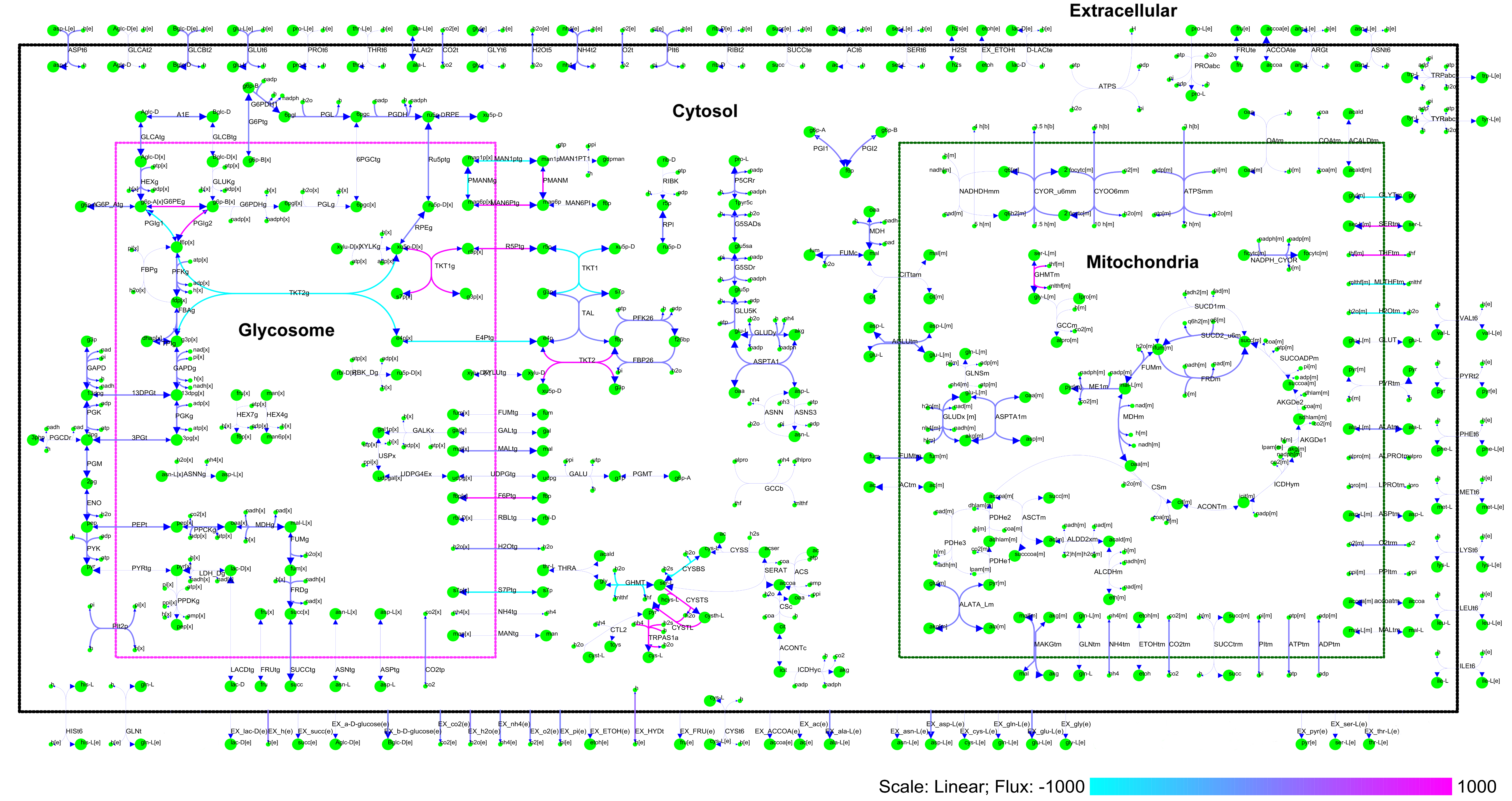

Supplement: S3 Fig — Flux distribution overlaid on pathway map. The map distinctly shows that glycolysis, pentose phosphate pathways and glutamate biosysnthesis reactions are constitutively activated in amastigote metabolism but, the reaction fluxes are considerably reduced as compared to promastigote metabolism. Also, there is reduced rate of ATP synthesis, glucose uptake and uptake of non-essential amino acids. (TIF) [file pone.0137976.s003.tif]

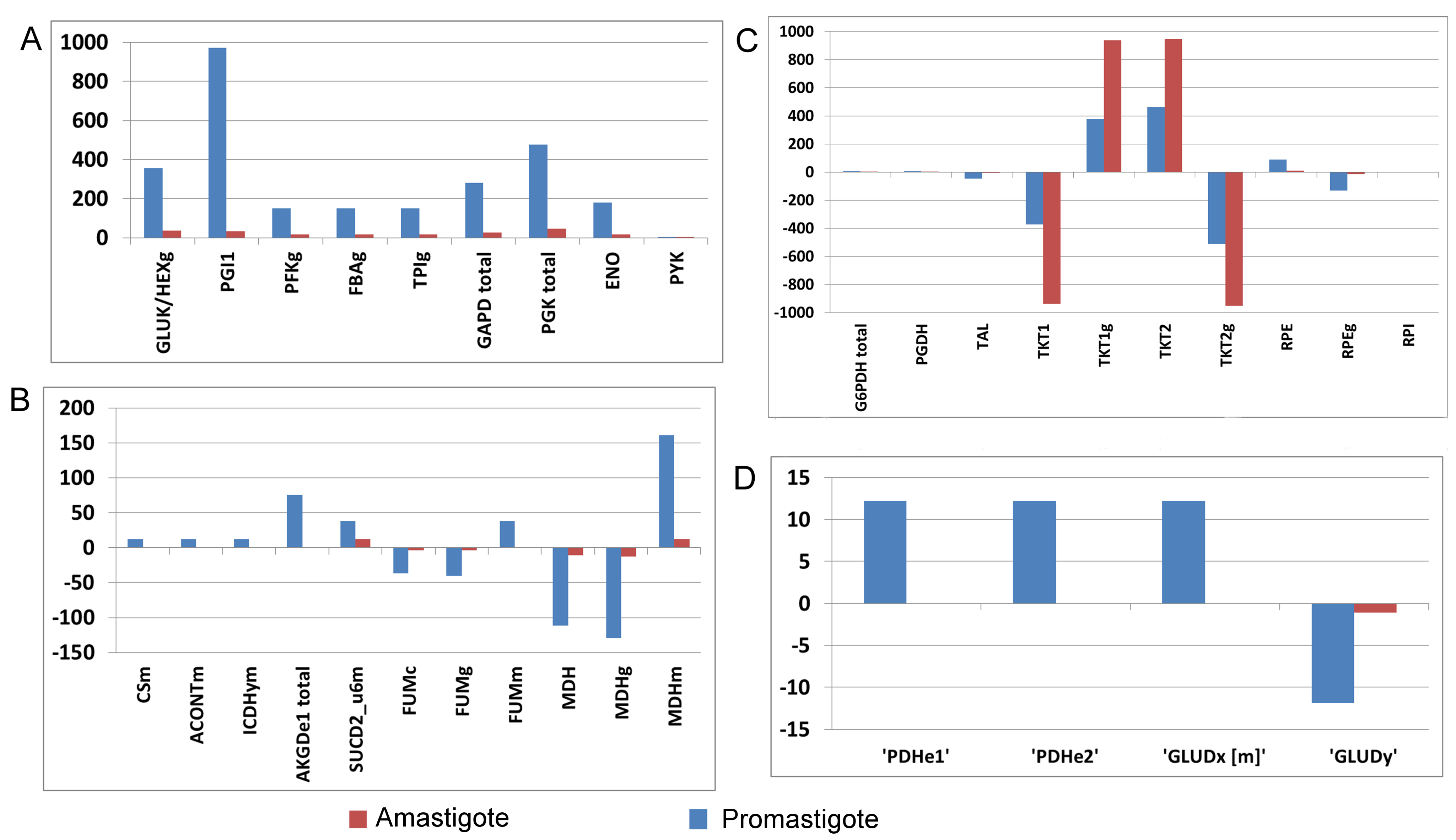

Supplement: S4 Fig — Bar plots showing differences between the amastigote and promastigote specific metabolism at the pathway level. A) differences observed in the glycolytic reactions in the amastigote and promastigote stages B) differences observed in the TCA cycle reactions in the two stages C) differences in the pentose phosphate pathway observed between the two stages D) differences between other important reactions in both the stages (TIF) [file pone.0137976.s004.tif]
