# Supplementary material for: Dissecting Leishmania infantum Energy Metabolism - A Systems Perspective
Source: PLoS One. 2015 Sep 14;10(9):e0137976. doi: 10.1371/journal.pone.0137976 (PMC4569355; doi:10.1371/journal.pone.0137976)
Supplement: S1 Text — Section A discusses about the formulation of the biomass objective function from 13C isotopic enrichment data. The other sections relatively support the results of the model described in the main article. It also contains three tables, Table SB, Table SC and Table SD, which are relevant to support the model analyses discussed in the methods and results section of main article. (DOC) [file pone.0137976.s012.doc]

**S1 Text**

**Section A**

**Annotation by structural analysis**

In case of aldose-1-epimerase, there was an ambiguity in assigning a substrate-level function to it. There are two copies of *Leishmania infantum* aldose-1-epimerase protein - UniProt IDs: A4I082, A4IAA4. We analyzed both the protein sequences for the presence of substrate specificity, if any. A glucose-6-phosphate epimerase (G6PE) was recently annotated in Yeast through structural studies (PDB ID = 2CIR) [1]. Using PyMol [2], the residues interacting with the phosphate moiety of glucose-6-phosphate in the 2CIR structure was identified. Structural analysis indicated that Arg-56 (RGGI stretch in protein sequence) and Arg-86 (RNST stretch in sequence) were in close proximity of the phosphate moiety of the substrate glucose-6-phosphate, providing an electrostatic interaction to the phosphate group. We procured the sequence of the PDB structure of yeast G6PE and performed a pairwise sequence alignment with both the aldose epimerase genes of *L. infantum*. To our interest, we found that the aforementioned sequence stretches were conserved in the aldose-1-epimerase sequence A4IAA4 (R=60, RGGV; R=84, RIRS). Whereas, the other sequence A4I082 did not show the presence of the amino acid stretches that were present in the yeast glucose-6-phosphate epimerase sequence. The conservation of the two arginine residues in one of the Aldose epimerase (A4IAA4) strongly indicated its substrate specificity towards glucose-6-phosphate. Hence, we suggest annotation of A4IAA4 as a “putative glucose-6-phosphate epimerase”.

**Confidence score**

To pinpoint the reliability of every reaction considered in the model, we propose a qualitative confidence score and assign it to every reaction in S1 Table. The confidence score is a five point score that unequally weighs the reliability of each model reaction with respect to the subcellular location information obtained from literature and predictions from sequence analysis. This Five point-scale is decided by three metrics, i) literature supporting the localization of a reaction, ii) Confirmation through sequence analysis using ScanProsite [3], and iii) Confirmation through sequence analysis using TargetP [4]. If and only if a literature finding clearly suggests about localization of a reaction, an arbitrary higher weight to the score (3) is given to that reaction. If the location of the enzyme and thus, the reaction was predicted solely through sequence analysis using ScanProsite and TargetP, then a score of 1 for each was assigned to that reaction. Clearly, if the enzyme location could be identified with appropriate literature evidence and also through predictions by ScanProsite and TargetP; the confidence score of that reaction would be the sum of the above individual scores i.e. 5 (3 + 1 +1). Thus, the highest score possible for a particular reaction would be 5 and the lowest score would be 1 depending upon the available information.

**The biomass objective function**

The biomass reaction (Biomass_iAS142) considered in the iAS142 model signifies a metabolic demand reaction that represents the drain of specific metabolites considered in the model. The coefficient of the metabolites in the biomass reaction was derived from 13C isotopic enrichment data quantitated from *Leishmania mexicana* log phase promastigotes grown in a completely defined medium containing 13C labeled glucose [3]. Enrichment data for only those metabolites present in the iAS142 model were considered for creating biomass reaction. The enrichment values (given in mol percent) scaled between 0 and 1 were used as coefficients of the metabolites considered in the biomass.

The biomass reaction thus created is given as follows:

0.9183pg[c] + 0.6634 asp_L[c] + 0.8192 glu_L[c] + 0.6667 ala_L[c] + 0.4743 g6p_B[c] + 0.8861 f6p[c] + 0.8915 s7p[c] + 0.8308 ru5p_D[c] + 0.8905 2pg[c] + 0.8483 pep[c] + 0.4481 g3p[c] + 0.7255 mal_L[c] + 0.6452 fum[c] + 0.7038 succ[c] + 0.4743 g6p_A[c] + 0.029 pro_L[c] + 0.0054 ser_L[c] 🡪

**Section B**

**Subcellular location assignment through sequence-based prediction**

For 22 reactions, subcellular location were annotated solely through sequence-based prediction of cellular localization signals. TargetP [4] was used for predicting the presence of a mitochondrial target peptide within a protein sequence and ScanProsite [3] was used for predicting the presence of a PTS signal. Proteins for which the prediction was made solely through sequence prediction are listed in SB Table.

Before assigning a specific compartment to a particular reaction, further confirmation of sequence-based detections were performed by identifying sequence homologues and probing them for presence of mitochondrial and peroxisomal signal sequences (S2 Table). Precisely 11 enzymes were assigned to their appropriate subcellular locations with respect to localization signals detected in its closest homologues, giving a higher confidence about their location (Table SB). But for around 7 enzymes, the assignment of location was made purely based on the results obtained from sequence-based detection of subcellular location but with low confidence (Table SB).

**Table SB.** Assignment of sub-cellular location to every enzyme through sequence-based prediction alone

| **Gene/Enzyme Name** | **Gene Symbol** | **Presence of PTS1/PTS2 (ScanProsite)** | **Presence of mitochondrial target peptide (TargetP)** | **Cellular Location** | **Cellular location predicted in closest homologues (See S2 Table for detailed results)** |
| --- | --- | --- | --- | --- | --- |
| Lactate dehydrogenase | LINJ_29_0290 | √ | --- | Glycosome | Glycosome |
| Lactate dehydrogenase | LINJ_27_1940 | √ | --- | Glycosome | Glycosome |
| Fructose-1,6-bisphosphate aldolase | LINJ_36_1320 | √ | --- | Glycosome | Glycosome |
| 2-oxoglutarate dehydrogenase (lipoamide) | LINJ_36_3630 | --- | √ | Mitochondria | Mitochondria |
| 2-oxoglutarate dehydrogenase (lipoamide) | LINJ_27_0740 | --- | √ | Mitochondria | Mitochondria |
| Dihydrolipoamide S-succinyltransferase | LINJ_28_2600 | --- | √ | Mitochondria | Mitochondria |
| Ribulokinase | LINJ_36_0060 | √ | --- | Glycosome | Glycosome |
| Xylulokinase | LINJ_36_0280 | √ | --- | Glycosome | --- |
| Galactokinase | LINJ_35_2790 | √ | --- | Glycosome | Glycosome |
| UDP-sugar pyrophosphorylase | LINJ_17_1260 | √ | --- | Glycosome | --- |
| phosphomannomutase | LINJ_34_3580 | √ | --- | Glycosome | --- |
| Malic enzyme | LINJ_24_0790 | --- | √ | Mitochondria | Mitochondria |
| alanine aminotransferase | LINJ_12_0580 | --- | √ | Mitochondria | Mitochondria |
| glutamate dehydrogenase | LINJ_28_3140 | --- | √ | Mitochondria | --- |
| aspartate aminotransferase | LINJ_24_0370 | --- | √ | Mitochondria | Mitochondria |
| glutamine synthetase | LINJ_06_0370 | --- | √ | Mitochondria | --- |
| asparaginase | LINJ_36_4650 | √ | --- | Glycosome | --- |
| glycine/serine hydroxymethyltranferase | LINJ_28_2530 | --- | √ | Mitochondria | --- |

*Note: The subcellular location of the above 18 enzymes were predicted solely through protein sequence based detection of cellular localization signals (PTS/mitochondrial targeting peptide detection). The locations were further confirmed by detection of these signals in the closest homologues excluding homologues from Genus Leishmania (as identified by BLAST against the RefSeq protein database with E-value < 1e-10) of the above enzymes.* [ ***√ :*** *indicates presence of that enzyme in that particular subcellular location;* ***--- :*** *indicates the absence of that enzymes in that particular subcellular location*]

**Section C**

**Comparison of knockout predictions between the *L. infantum* iAS142 and *L. major* iAC560 models**

Given below is a table (Table SC) that compares the knockout predictions of the iAS142 with the iAC560 model lethal predictions and their relation to known phenotypes identified from experiments. The results suggest that the iAS142 model can accurately predict the actual phenotypes identified through known knockout experiments in comparison to iAC560. These differences are primarily due to differences in reaction network topology/structure between the iAS142 and iAC560 models.

Flux balance analysis of the iAS142 network identifies a subset of tightly coupled reactions that form part of glucose and non-essential amino acid metabolism, which is unique to iAS142 and not observed in other Trypanosomatid models. The subset that maximizes biomass under different carbon sources represents a common metabolic route from glucose uptake -> succinate fermentation -> TCA cycle -> Glutamate biosynthesis. 13C isotopomer profiling studies in *Leishmania mexicana* [5] have also identified the same metabolic route as identified from the iAS142 model predictions. This reaction subset would probably remain conserved even if reactions from other pathways are included within the iAS142 network as they represent a tightly balanced subset which satisfies ATP and redox balance within different compartments and thereby maximizes the biomass. Reactions that form a part of this sub-network were predicted to govern a lethal phenotype by knockout analysis in iAS142. The above metabolic route was not observed in *L. major* iAC560 model and hence, the lethality predictions of the iAC560 model do not match with experimentally known phenotypes. The reasons for these differences between models and its implications on model predictions have further been discussed in results section of the main article.

**Table SC: Comparison of knockout phenotypes predicted from iAS142 and iAC560 models**

| **Reaction name** | ***L. major* iAC560 predictions** | ***L. infantum* iAS142 predictions** | **Experimental phenotype references** |
| --- | --- | --- | --- |
| ACONTm | Non-Lethal | Lethal | Lethal |
| ATPSmm | Lethal | Lethal | Lethal |
| CYOO6mm | Non-Lethal | Lethal | Lethal |
| FBPg | Non-Lethal | Non-Lethal | Non-Lethal |
| MAN6PI | Lethal | Non-Lethal | Non-Lethal |
| PMANM | Lethal | Non-Lethal | Non-Lethal |
| USPx | Not there in model | Non-Lethal | Non-Lethal |

**Section D**

**Comparison of growth phenotype information between *Leishmania* *infantum* and other Trypanosomatids**

For comparison of the predicted growth phenotypes with other Trypanosomatids, we restricted to the insect-stage procyclic *T. brucei* phenotype information [6-10], as the promastigote and *T. brucei* insect stages exist in fly midgut. These differences could probably be observed because the insect stages of *Trypanosoma* live in a micro-environment that is different from the *Leishmania* species and also, because of the different genotypes of these two species. The comparisons are given in Table SD.

**Table SD: Comparison of the model-predicted growth phenotypes with experimentally known growth phenotypes of other *Trypanosoma*** species

| **Model reaction constrained** | **Experimental Target** | **Model prediction** | **Experimental finding** | **Reference (for experiment)** | **Organism** | **Predicted wild type growth (%)** |
| --- | --- | --- | --- | --- | --- | --- |
| ACONTm | Aconitase | lethal | nonlethal | [6] | *T. brucei* | 0 % |
| PGK | Phosphoglycerate kinase | nonlethal | lethal | [7] | *T.brucei* | 99.99 % |
| PYK | Pyruvate kinase | nonlethal | lethal | [8] | *T. brucei* | 99.99 % |
| SUCOADPm | Succinyl CoA synthetase | nonlethal | lethal | [9] | *T. brucei* | 99.99 % |
| UDPG4Ex | UDP-glucose/galactose 4-epimerase | nonlethal | lethal | [10] | *T. brucei* | 99.99 % |

**Section E**

**Effect of amino acid uptake when supplemented with glucose**

*L.mexicana* developmental stages were shown to co-utilize glucose and a few, selected non-essential amino acids like glutamate, aspartate, alanine and proline, when cultured in a completely defined medium containing a range of carbon sources [5],[11]. In order to understand the role played by every non-essential amino acid, the flux distribution was observed by supplementing the glucose uptake with uptake of non-essential amino acids like aspartate, proline, glutamate, and alanine, one at a time. Detailed information of the same can be obtained from the Results section of the main article. For obtaining an optimum biomass, the optimal value attained by the uptakes of individual amino acids when supplemented with glucose uptake were in the order – proline uptake < aspartate uptake < glutamate uptake < alanine uptake (see Panel A in S1 Fig). By opening one amino acid uptake at a time along with glucose uptake and performing FBA for each situation, the optimum for biomass production/growth rate was obtained in the order – glutamate uptake > alanine uptake > aspartate uptake > proline uptake (see Panel B in S1 Fig).

**Section F**

**Comparison of energy metabolism between different developmental stages of *L.infantum***

Amastigotes display a reduced ATP synthesis and hence, a reduced growth rate as compared to promastigotes [11]. Also, the uptake rates of glucose and non-essential amino acids are highly reduced in the amastigote scenario as compared to the promastigote [5], [11]. From the model, we recreated scenarios for promastigote and amastigote metabolism and found out differences in metabolism that are intuitive with respect to the environment in which the parasite resides in (see S2 and S3 Figs). Detailed information on the stage specific metabolic differences observed from the model can be obtained from the Results section of the main article. The change in the reaction fluxes of these pathways relate to the change in the specific activity of certain crucial enzymes that differ between the promastigote and amastigote forms of the parasites [12]. We monitored the differences between the fluxes of reactions catalyzed by these enzymes in the two stage specific scenarios created to analyze if we could identify the same differences (see S4 Fig). Panel A in S4 Fig demonstrates the differences observed in the glycolytic reactions in the two stages. It could be seen that flux through glycolysis is largely reduced in amastigote as compared to promastigote due to reduced influx of glucose into the cell. Panel B in S4 Fig demonstrates the differences in the TCA cycle reactions in the two stages. The flux through TCA cycle is highly reduced in the amastigote as compared to the promastigote scenario. Panel C in S4 Fig demonstrates the differences in the pentose phosphate pathway observed between the two stages. Flux through all other reactions except the transketolase reactions was quite similar in both the stages even though comparatively, flux through pentose phosphate pathway was reduced in case of amastiogtes. In glucose-deficient conditions, the demand for glucose-6-phosphate is largely increased. This requirement is fulfilled by the pentose phosphate pathway in case of the amastigotes. Panel D in S4 Fig demonstrates the differences between other important reactions in both the stages. The fluxes through pyruvate dehydrogenase and glutamate dehydrogenase reactions were quite low in amastigotes as compared to promastigotes.

**REFERENCES**

1. Graille M, Baltaze J-P, Leulliot N, Liger D, Quevillon-Cheruel S, van Tilbeurgh H, et al. Structure-based functional annotation yeast ymr099c codes for a D-hexose-6-phosphate mutarotase. J Biol Chem. 2006; 281: 30175–30185

2. DeLano WL PyMOL. DeLano Scientific, San Carlos, CA 700, 2000.

3. De Castro E, Sigrist CJA, Gattiker A, Bulliard V, Langendijk-Genevaux PS, Gasteiger, E, et al. ScanProsite: detection of PROSITE signature matches and ProRule-associated functional and structural residues in proteins. Nucleic Acids Res. 2006; 34: W362–W365.

4. Emanuelsson O, Brunak S, von Heijne G, Nielsen H. Locating proteins in the cell using TargetP, SignalP and related tools. Nat Protoc. 2007; 2: 953–971.

5. 11. Saunders EC, Ng WW, Chambers JM, Ng M, Naderer T, Kromer JO, et al. Isotopomer profiling of *Leishmania mexicana* promastigotes reveals important roles for succinate fermentation and aspartate uptake in tricarboxylic acid cycle (TCA) anaplerosis, glutamate synthesis, and growth. J Biol Chem. 2011; 286: 27706–27717.

6. Van Weelden SWH, Fast B, Vogt A, van der Meer P, Saas J, van Hellemond JJ, et al. Procyclic *Trypanosoma brucei* do not use Krebs cycle activity for energy generation. J Biol Chem. 2003; 278: 12854–12863.

7. Bressi JC, Choe J, Hough MT, Buckner FS, Van Voorhis WC, Verlinde CLMJ, et al. Adenosine analogues as inhibitors of *Trypanosoma brucei* phosphoglycerate kinase: elucidation of a novel binding mode for a 2-amino-N(6)-substituted adenosine. J Med Chem. 2000; 43: 4135–4150.

8. Coustou V, Besteiro S, Biran M, Diolez P, Bouchaud V, Voisin P, et al. ATP generation in the *Trypanosoma brucei* procyclic form: cytosolic substrate level is essential, but not oxidative phosphorylation. J Biol Chem. 2003; 278: 49625–49635.

9. Bochud-Allemann N, Schneider A. Mitochondrial substrate level phosphorylation is essential for growth of procyclic *Trypanosoma brucei*. J Biol Chem. 2002; 277: 32849–32854.

10. Roper JR, Guther MLS, Milne KG, Ferguson MAJ. Galactose metabolism is essential for the African sleeping sickness parasite Trypanosoma brucei. Proc Natl Acad Sci U.S.A. 2002; 99: 5884–5889.

11. Saunders EC, Ng WW, Kloehn J, Chambers JM, Ng M, McConville MJ. Induction of a stringent metabolic response in intracellular stages of *Leishmania mexicana* leads to increased dependence on mitochondrial metabolism. PLoS Pathog. 2014; 10: e1003888.

12. Meade JC, Glaser TA, Bonventre PF, Mukkada AJ. Enzymes of Carbohydrate Metabolism in *Leishmania donovani* amastigotes. J Protozool. 1984; 31: 156–161.
